# Photogrammetry and 3D Gaussian Splatting for 4D plant growth capture and greenhouse digital twins

**DOI:** 10.3897/BDJ.14.e191083

**Published:** 2026-10-23

**Authors:** Toivo Aukusti Ylinampa, Urmas Kõljalg

**Affiliations:** 1 University of Tartu, Tartu, Estonia University of Tartu Tartu Estonia

**Keywords:** 3D digitisation, 3D Gaussian Splatting, 4D phenotyping, botanical garden, Multi-View Stereo, Structure-from-Motion, visualisation

## Abstract

Plants are inherently three-dimensional organisms, yet many biologically important traits remain difficult to document using two-dimensional photography because of self-occlusion and complex plant architecture. Image-based three-dimensional (3D) digitisation offers an accessible alternative using standard RGB cameras, while recent advances in 3D Gaussian Splatting (3DGS) enable photorealistic real-time visualisation of reconstructed scenes.

We present a practical workflow that combines Structure-from-Motion/Multi-View Stereo (SfM/MVS) photogrammetry with 3D Gaussian Splatting for botanical digitisation across multiple spatial scales. The workflow was evaluated using a 12-timepoint ("4D") fern growth series, a greenhouse digital twin assembled from fourteen independently reconstructed sections and additional datasets including a smartphone-photographed flower, two macro-imaging workflows, a potted plant, a drone-based tree reconstruction and a controlled wind experiment. Global multi-day image alignment was used to establish a common coordinate system for the fern time series, allowing individual daily reconstructions to share the same scale and orientation without repeated fixed camera positions.

Across the fern time series, photogrammetric reconstructions produced 95,698–325,415 tie points with RMS re-projection errors of 1.36–1.72 pixels. Corresponding 3DGS models achieved PSNR values of 21.99–26.35 dB, SSIM values of 0.726–0.842 and LPIPS values of 0.223–0.339. Controlled macro-imaging produced the highest perceptual quality, whereas a dedicated wind experiment demonstrated a marked reduction in reconstruction fidelity caused by leaf movement. The greenhouse sections were manually assembled into an interactive digital twin suitable for visual exploration, although not intended as a metrically validated spatial survey.

The study demonstrates that consumer-grade cameras, photogrammetry and 3D Gaussian Splatting support reproducible botanical digitisation from macro-scale flowers to greenhouse environments. The presented workflow provides practical guidance for generating scalable 3D datasets for plant documentation, living collections, digital exhibitions and future quantitative analyses.

## Introduction

Plants are inherently three-dimensional organisms and many biologically and taxonomically relevant traits — such as branching architecture, phyllotaxis, leaf curvature and flower structure — can be difficult to observe from two-dimensional (2D) images due to self-occlusion and viewpoint- and projection-dependent appearance. This has increased interest in 3D digitisation methods that: (1) preserve complex plant morphology in a shareable format; (2) enable quantitative measurements when acquisition conditions and scale are controlled and (3) support digital documentation of living collections and biodiversity.

**Structure-from-Motion photogrammetry in plant morphology**

Image-based photogrammetry, especially Structure-from-Motion (SfM) combined with Multi-View Stereo (MVS), is widely used for plant 3D reconstruction from overlapping RGB images (Pound et al. 2014). SfM estimates camera poses and a sparse point cloud, while MVS densifies the reconstruction to generate dense point clouds and, in many workflows, textured surface meshes. In plant phenotyping, SfM/MVS has been used to estimate traits, such as plant height, leaf length and canopy volume and can achieve strong agreement with reference measurements under suitable conditions. For example, Herrero-Huerta et al. (2023) and Li et al. (2024) reported coefficients of determination (R²) > 0.90 when SfM/MVS-derived traits for wheat and maize were compared against ground-truth measurements.

SfM has also been used in ecological and forestry applications, where it can provide a lower-cost alternative to LiDAR for selected measurements. Iglhaut et al. (2019) reviewed SfM usage in forestry, including applications to tree height and biomass estimation. More broadly, geometric 3D approaches are non-destructive (Paulus 2019), but extracting robust phenotypic information from 3D models remains challenging (Harandi et al. 2023). A key advantage of photogrammetric reconstruction is its versatility across plant sizes and environments. Andújar et al. (2018) reconstructed weeds, while Wu et al. (2020) and Wu et al. (2022) developed portable multi-view workflows for maize and smaller plants. For certain traits and conditions, photogrammetry can be competitive with laser scanning (Wang et al. 2018). However, reconstruction quality is sensitive to acquisition conditions: wind-driven motion and changing illumination can reduce feature matching and, thus, degrade reconstruction accuracy (Paturkar et al. 2020). At broader spatial scales, image-based methods have also been applied to canopy structure reconstruction (Hui et al. 2018), indicating potential for ecological and agronomic research.

**3D Gaussian Splatting as a new tool for visualisation and digital twins**

Neural rendering has recently emerged as a complementary tool for plant digitisation and phenotyping (Akhtar et al. 2024). 3D Gaussian Splatting (3DGS; Kerbl et al. (2023)) represents scenes as a set of anisotropic Gaussian primitives (“splats”), optimised from multi-view imagery and rendered in real time. Compared to mesh-based reconstruction, 3DGS can provide photorealistic visualisation and may be advantageous in scenes with thin foliage, complex occlusion and view-dependent reflectance. Stuart et al. (2025) demonstrated 3DGS reconstruction for wheat and reported an average reconstruction error of ~ 0.74 mm in their experimental setting. Ojo et al. (2024) also demonstrated plant capture using Gaussian splatting, supporting its promise as an accessible representation for interactive visualisation. Beyond crop plots, 3DGS has been applied to trees and larger outdoor scenes, including leafless tree branch reconstruction (Li et al. 2025) and drone-based forestry mapping (Shaheen et al. 2025), highlighting its potential for multi-scale scene digitisation.

**4D digitisation: multi-temporal reconstruction and growth modelling**

Multi-temporal image-based reconstruction has been used to create “4D” time series for characterising plant growth dynamics. Using sequential reconstructions, Duan et al. (2016) measured wheat growth over time, while EasyDCP provides an open-source phenotyping pipeline that can approach the performance of commercial laser-scanning alternatives for certain tasks (Feldman et al. 2021). At field scale, (Malambo et al. (2018) used UAV SfM imaging to generate maize and sorghum time series. Nevertheless, scaling multi-temporal imaging remains challenging: Kobe et al. (2024) reported issues with image blur and scaling stereo approaches to larger barley-field areas and Ma et al. (2025) showed that while time-series trait tracking in greenhouse lettuce is feasible with MVS, inner-leaf occlusion and high computational requirements can be limiting. Together, these studies indicate that 4D phenotyping with geometric reconstruction is feasible, but often constrained by occlusion, motion and computational cost. Recent work extends 3DGS towards temporal plant visualisation and digital twin applications. Shen et al. (2025) introduced PlantGaussian for cross-time plant visualisation using 3DGS and Adebola et al. (2025) introduced GrowSplat, combining 3DGS reconstruction with spatial alignment to create 4D growth models. These developments suggest that combining geometric reconstruction and neural rendering may provide a practical route to multi-temporal digitisation that supports both analysis and communication.

**Plant digitisation for collections, taxonomy and communication**

In addition to phenotyping, 3D digitisation is increasingly used for documentation of collections and for education and outreach. Carpological collections have been digitised (Wang et al. 2022) and flower structures have been reconstructed using accessible DSLR-based workflows (Leménager et al. 2022). At the scale of living collections, digital botanical garden projects suggest that integrating plant models into interactive environments can enhance visitor experience and support collection management (Redweik et al. 2022). Point-cloud-based VR experiences have also been used to visualise botanical gardens (Rubin et al. 2023). Collectively, these works suggest that 3D representations can support scientific workflows and public communication, provided that methods and outputs are documented in a reproducible manner.

**Aim of this study**

Although photogrammetry and 3D Gaussian Splatting (3DGS) have advanced rapidly, existing botanical applications often focus on specialised phenotyping systems, single reconstruction techniques or individual case studies. Relatively few studies describe a complete, low-cost workflow that can be applied across multiple spatial scales using consumer-grade cameras, while supporting multi-temporal ("4D") reconstruction.

The aim of this study was to develop and evaluate a practical workflow that integrates Structure-from-Motion photogrammetry and 3D Gaussian Splatting for botanical digitisation. Specifically, we demonstrate the workflow using: (1) a 12-timepoint ("4D") fern growth series reconstructed within a common coordinate system; (2) a multi-part digital twin of a tropical greenhouse assembled from fourteen independently reconstructed sections; and (3) additional case studies spanning macro, handheld, drone and imaging scenarios. We further evaluate reconstruction quality using photogrammetric and neural-rendering metrics, examine practical factors affecting reconstruction performance, including wind and acquisition geometry and provide reproducible acquisition guidelines, workflow diagrams and supplementary capture-path visualisations to facilitate adoption by the botanical and natural history communities.

## Materials and Methods

AI usage: Grammarly was used to proofread this manuscript.

**Study sites, specimens and imaging equipment**

**Figure 1.**
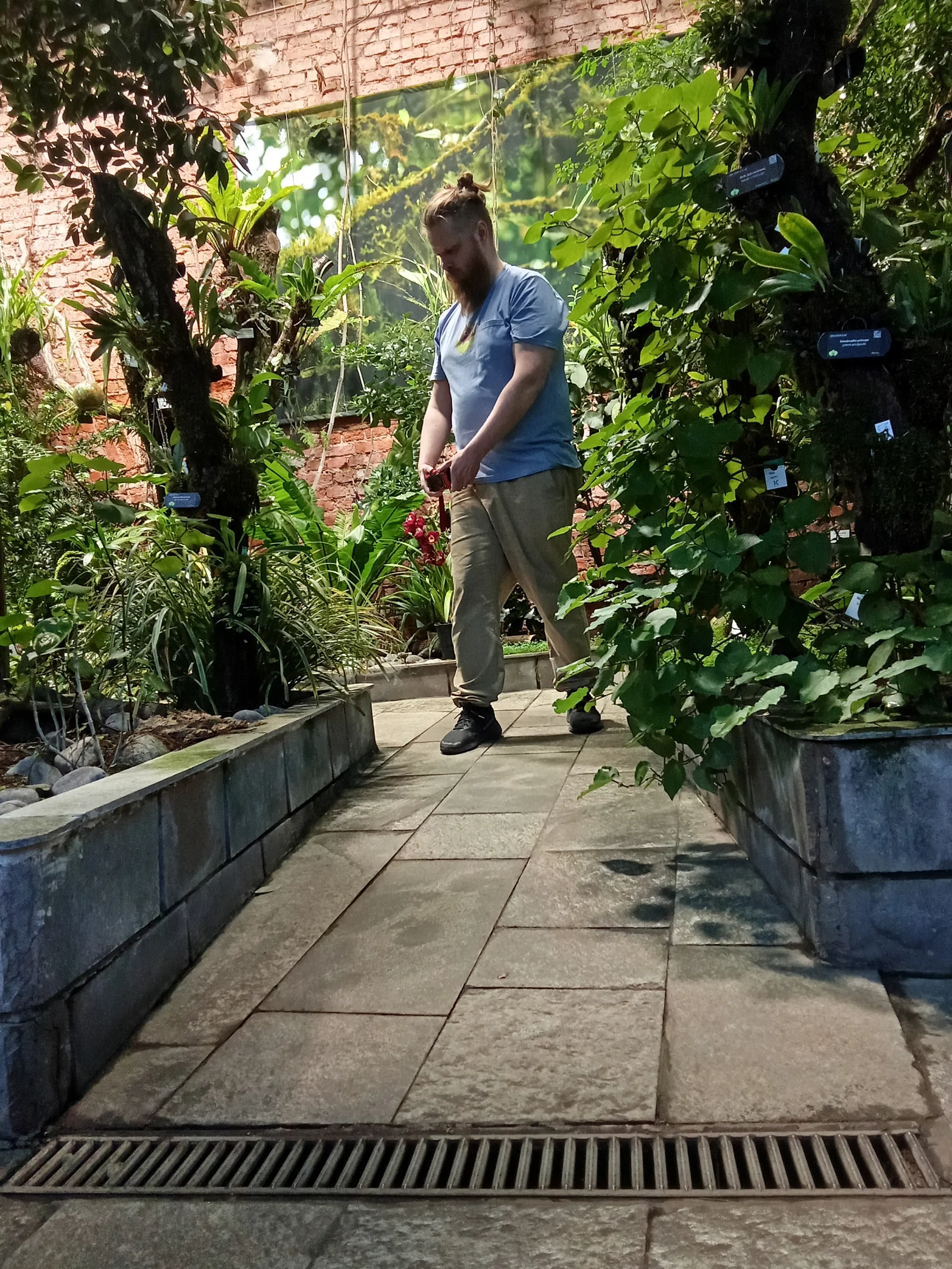
Greenhouse capture with an Olympus TG-6.

A 12-timepoint ("4D") time series of a fern was captured outdoors at the University of Tartu Botanical Garden. In addition, the tropical greenhouse of the same garden was digitised as fourteen spatially overlapping sections. Both datasets were acquired using an Olympus TG-6 compact camera (12 MP; Fig. 1). Outdoor imaging was performed near mid-day during calm, rainless weather to minimise illumination changes and wind-induced motion.

To evaluate the proposed workflow across different spatial scales, imaging conditions and acquisition scenarios (Table 1), additional datasets were collected: (i) a potted plant photographed using an Olympus TG-6; (ii) a greenhouse flower photographed using a Xiaomi Redmi A3 smartphone (8 MP); (iii) an *in situ* macro dataset acquired from an indoor plant wall using a Canon EOS 70D, fitted with a Canon EF 100 mm f/2.8 Macro lens mounted on a tripod under portable studio lighting ("MacroWall"); (iv) a controlled macro dataset acquired using the same camera and lens with the specimen positioned on a portable hexagonal calibration marker to enable metric scaling ("MacroHexagon"); (v) a drone-based tree reconstructed from imagery captured with a DJI Mavic Air 2; and (vi) an indoor wind experiment in which the same fern was photographed under both stationary and artificially induced wind conditions to evaluate the effect of plant motion on reconstruction quality.

**Image acquisition workflow**

The workflow was designed to be portable using consumer-grade equipment. Images were acquired manually, except for the MacroWall dataset, which used a tripod to maximise image sharpness. Three battery-powered softbox lights (5500K) provided diffuse illumination for macro photography.

Camera settings were kept constant within each image set where practical. Although RAW images are generally preferred for photogrammetry, all datasets were reconstructed successfully from standard JPEG images.

Images were captured from multiple viewpoints with high overlap to minimise self-occlusion. When metric reconstruction was required, reference distances were introduced into the models. For the fern time series, stable distances in the surrounding environment were measured and implemented as scale bars in Agisoft Metashape. MacroHexagon was scaled using a portable hexagonal calibration marker with high-contrast feature points; opposing marker vertices were measured using digital calipers before image acquisition. MacroWall was scaled by directly measuring a stable floral structure with digital calipers and introducing the same distance as a Metashape scale bar. An independent measurement performed on the reconstructed model was then compared with the physical caliper measurement to verify scaling accuracy.

Dataset size was limited primarily by system memory. Using a workstation with 48 GB RAM, approximately 2,000 images could be aligned using High alignment accuracy, whereas larger datasets required either Low alignment accuracy or subdivision into smaller projects.

**Photogrammetric reconstruction (SfM/MVS)**

Photogrammetric processing was performed in Agisoft Metashape Professional v.2.2.2 on a Windows workstation (Intel i7-8086K 4.0 GHz, 48 GB RAM, NVIDIA GTX 1080 6 GB). Source images were stored as JPEG (.JPG) files and projects saved in PSX format.

Sparse alignment was computed using High accuracy for all datasets, except the fern time series (~ 8,500 images), which was aligned using Low accuracy owing to its size. Mesh and texture generation used High quality settings unless otherwise stated.

**Cross-time alignment of the fern time series**

**Figure 3.**
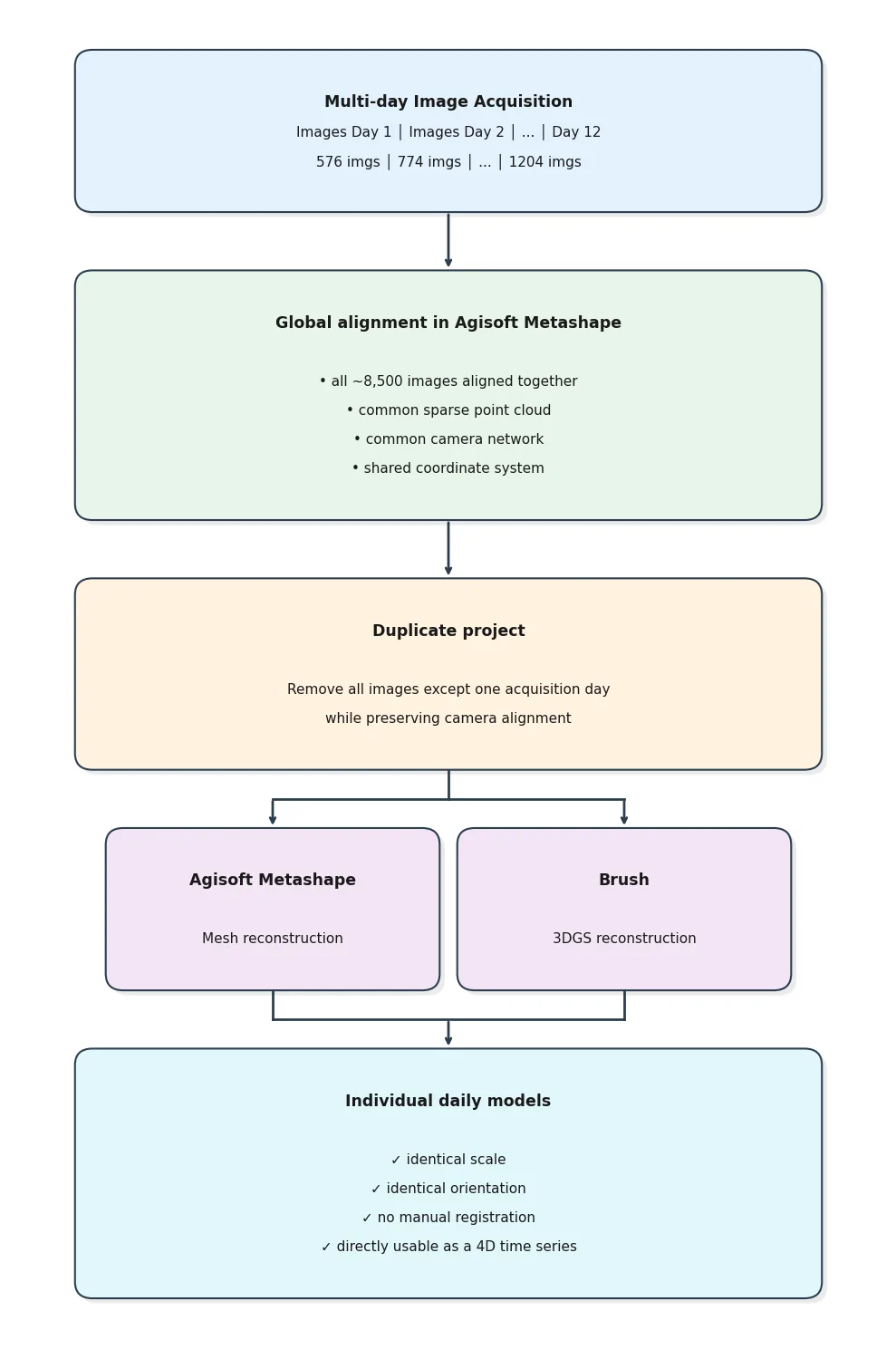
4D workflow.

To establish a common coordinate system across all twelve timepoints, all fern images were first aligned within a single Metashape project (Fig. 2and Fig. 3), producing a shared sparse point cloud and camera network constrained by stable background features. The project was then duplicated into twelve individual projects by retaining only the images from a single day, while preserving the original alignment.

This procedure enabled every daily reconstruction to inherit identical orientation and scale without requiring repeated camera positions. After scaling the global alignment, all derived reconstructions shared the same metric coordinate system, allowing direct temporal comparison.

**3D Gaussian Splatting reconstruction**

Camera calibration, camera poses, sparse point clouds and source images were exported from Metashape in COLMAP format (cameras.txt, images.txt, points3D.txt and associated image files) and imported into Brush v.0.3 running on a Mac Studio (32 GB RAM). Brush was selected because it provides native macOS support, imports COLMAP projects directly from Metashape, implements standard 3D Gaussian Splatting optimisation and reports quantitative metrics (PSNR, SSIM and LPIPS). Models were trained for 30,000 optimisation iterations. Resulting Gaussian splats were edited in SuperSplat v.2.10 to remove floating splats and background artefacts before being imported into Unity (v.6000.0.23f1) using the UnityGaussianSplatting plugin (v.1.1.1). The fourteen greenhouse sections were manually assembled using overlapping visual features to create an interactive digital twin.

**Quantitative evaluation**

Photogrammetric quality was assessed using the number of tie points and RMS re-projection error reported by Metashape. Rendering quality of the 3DGS models was evaluated in Brush using a one-in-eight image hold-out protocol. Reconstruction quality was quantified using Peak Signal-to-Noise Ratio (PSNR), Structural Similarity Index Measure (SSIM) and Learned Perceptual Image Patch Similarity (LPIPS). These metrics compare rendered images with previously unseen photographs and, therefore, evaluate rendering fidelity rather than geometric accuracy.

**Capture-path visualisation and supplementary material**

To facilitate reproducibility without distributing tens of thousands of source images, supplementary material includes capture-path visualisations derived from reconstructed camera positions, demonstration videos of both photogrammetric and 3DGS models and workflow videos illustrating the global fern alignment procedure and preservation of the shared coordinate system.

## Results

**Quantitative reconstruction quality**

Quantitative reconstruction statistics are summarised in Tables 1, 2, 3 and Table 4. Photogrammetric quality was evaluated using the number of tie points and RMS re-projection error reported by Agisoft Metashape, whereas 3D Gaussian Splatting (3DGS) quality was assessed using PSNR, SSIM and LPIPS, calculated by Brush using a one-in-eight image hold-out protocol.

Reconstruction quality varied primarily with acquisition conditions. Controlled macro datasets produced the highest perceptual quality, whereas the drone-based tree yielded the lowest PSNR and SSIM values. The MacroHexagon dataset achieved the lowest LPIPS, while the indoor windless fern produced the highest PSNR and SSIM.

**Fern 4D time series**

**Figure 4.**
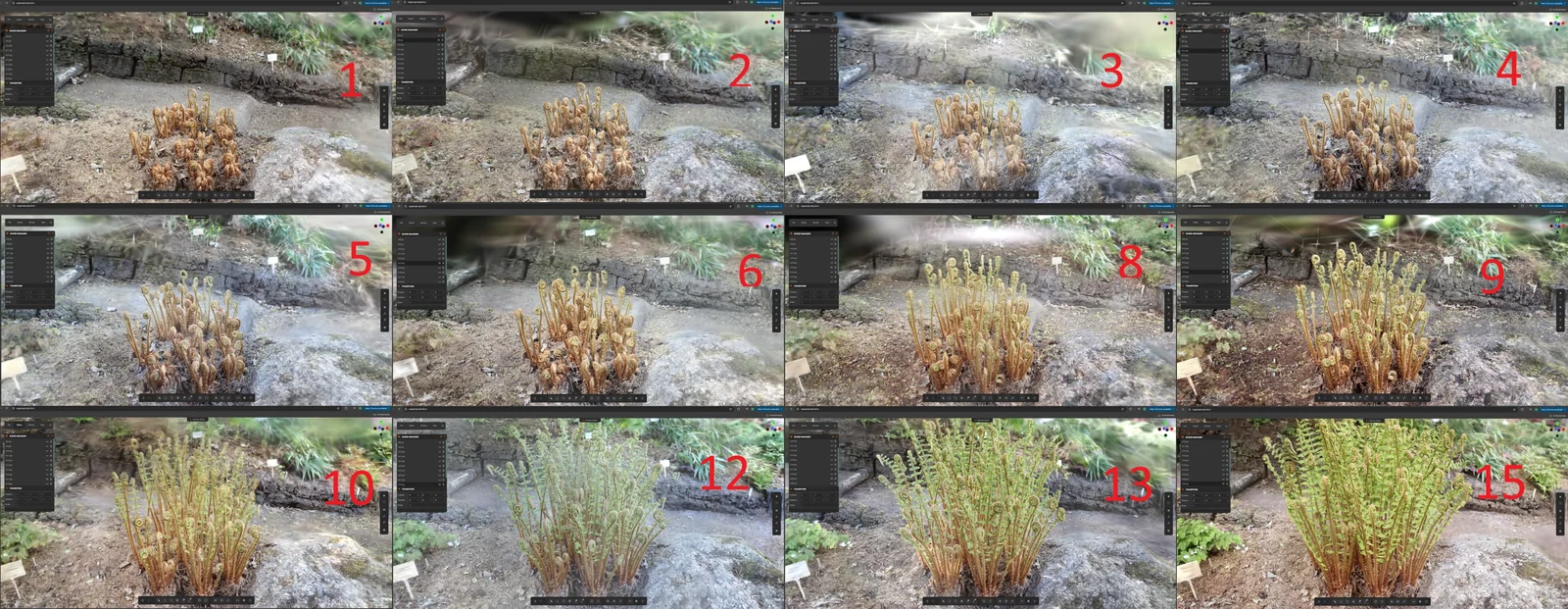
4D Fern growth timelapse, 12 3DGS reconstructions. Using Metashape's multi-day global alignment, all the resulting individual day datasets share common scale and orientation without the need for manual adjustments.

Approximately 8,500 images were acquired during twelve imaging sessions spanning fifteen days, with individual time points containing 341–1,204 photographs. All images were aligned simultaneously in a single Metashape project to establish a shared camera network and coordinate system before being separated into individual daily reconstructions (Fig. 4).

Photogrammetric reconstructions produced 95,698–325,415 tie points with RMS re-projection errors of 1.36–1.72 pixels (Table 2). Brush training required approximately three hours per time point using 30,000 optimisation iterations, producing PSNR values of 21.99–26.35 dB and SSIM values of 0.726–0.842. As all daily reconstructions shared the same coordinate system, they could be visualised directly as a 4D growth sequence without additional registration.

**Greenhouse digital twin**

The tropical greenhouse was reconstructed from fourteen independently acquired image sets comprising approximately 20,000 photographs. Individual sections contained 546–1,643 images. Some sections can be observed in figures below (Figs 5, 6, 7, 8, 9, 10, 11, 12, 13, 14, 15, 16). Top-left: Textured 3D model in Metashape. Top-right: camera positions. Bottom-left: Mesh confidense level in Metashape. Bottom-right: 3D Gaussian Splat.

Photogrammetric reconstructions produced 329,238–1,115,847 tie points with RMS reprojection errors between 1.49 and 2.35 pixels (Table 3). Brush evaluation produced PSNR values of 21.49–26.26 dB, SSIM values of 0.695–0.846 and LPIPS values of 0.310–0.520.

**Figure 17.**
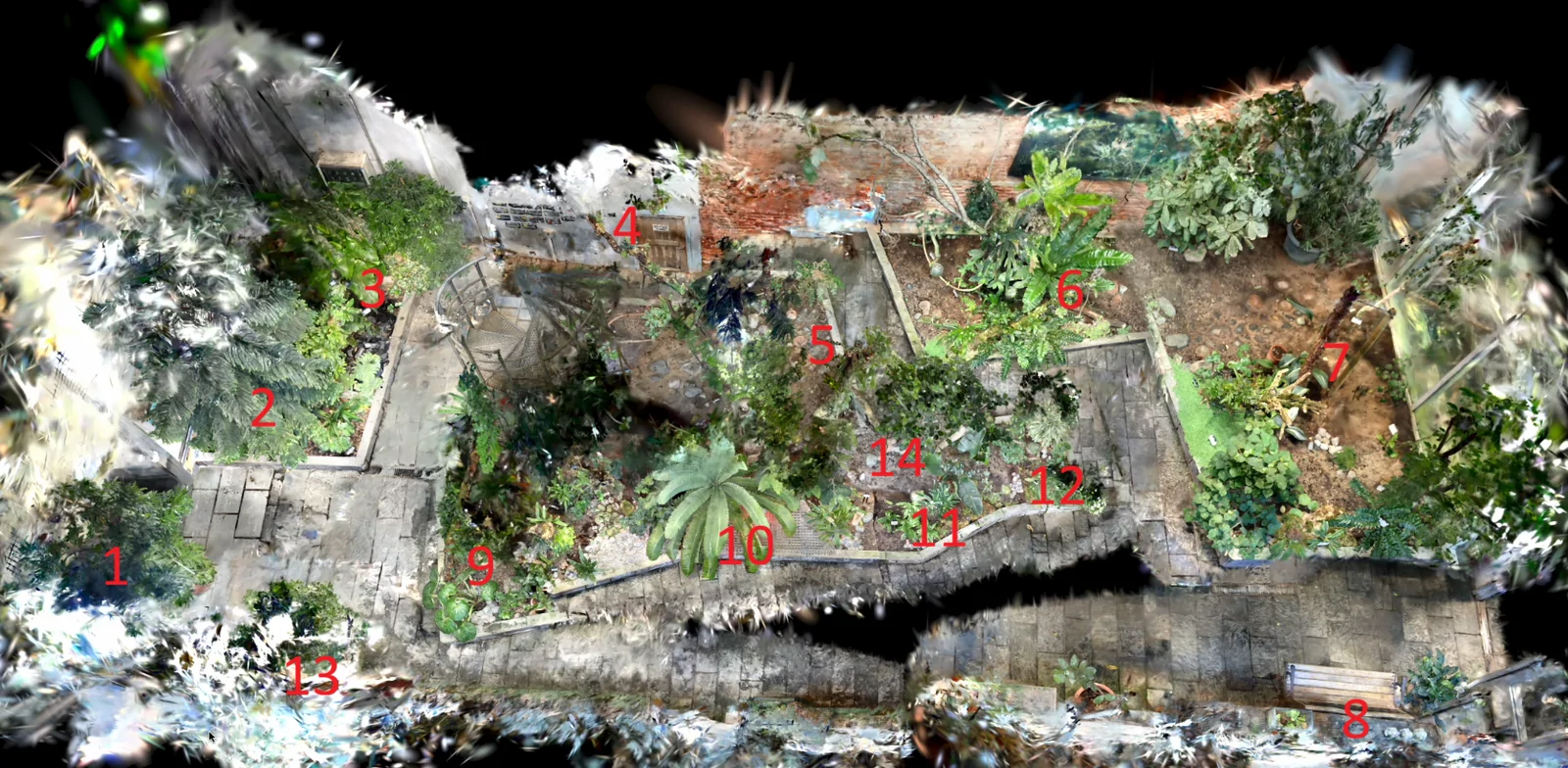
Overall view of 14 individual 3D Gaussian Splats, visualised in Unity Game Engine.

After editing in SuperSplat, the fourteen sections were assembled manually in Unity to create an interactive greenhouse digital twin (Fig. 17). As assembly was performed manually, the combined model is intended primarily for visual exploration rather than metric spatial analysis.

**Wind experiment**

**Figure 18.**
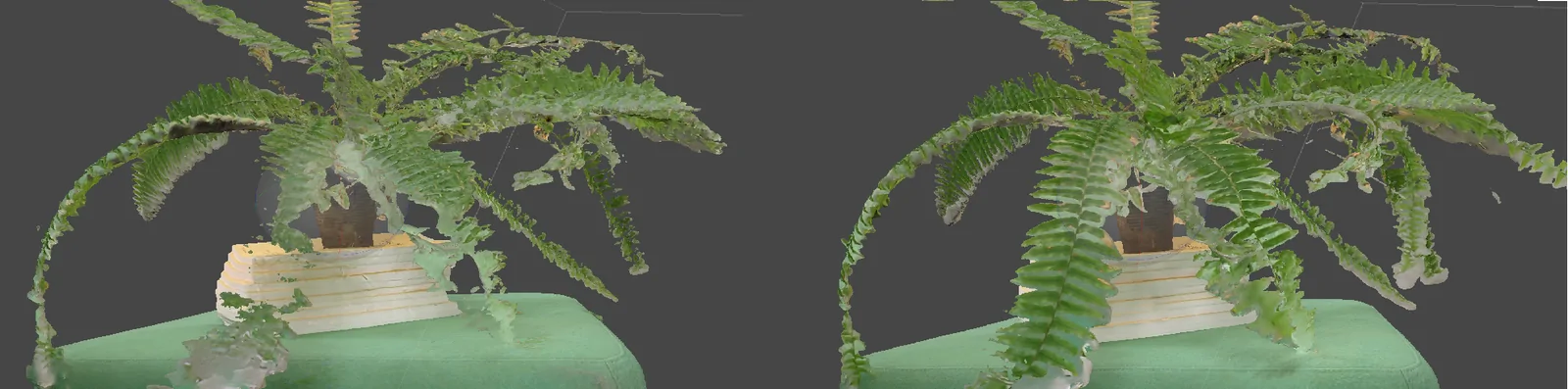
The effect of wind in 3D reconstruction. Windy conditions on the left resulted in inferior 3D reconstruction.

The effect of plant motion was evaluated using two indoor datasets acquired under identical camera settings, one with stationary foliage and one with artificially induced wind (Fig. 18).

Wind substantially reduced reconstruction quality. The stationary plant achieved higher PSNR and SSIM values and a lower LPIPS value than the moving plant (Table 3), demonstrating that even modest leaf movement degrades image-based reconstruction.

**Additional case studies**

The remaining datasets demonstrate the applicability of the workflow across imaging scales.

**Figure 19.**
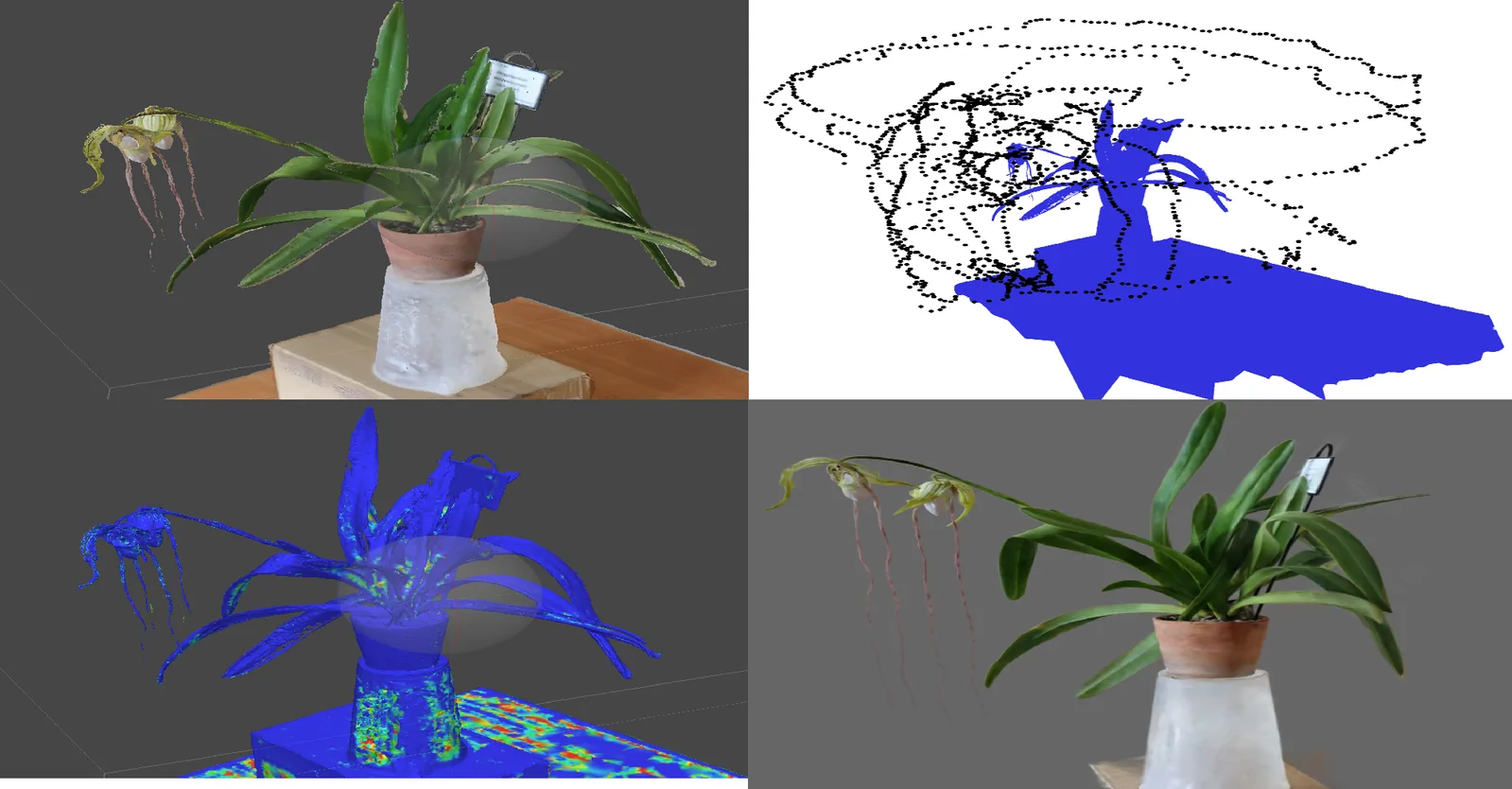
A potted plant. It is noteworthy that the hanging flower structucture was better reconstructed in 3DGS than in Photogrammetry.

The potted plant was successfully reconstructed using both photogrammetry and 3DGS. Long hanging floral structures were only partially reconstructed in the mesh model, but were reproduced almost completely by 3DGS, illustrating its advantages for thin, highly occluded plant structures (Fig. 19).

**Figure 20.**
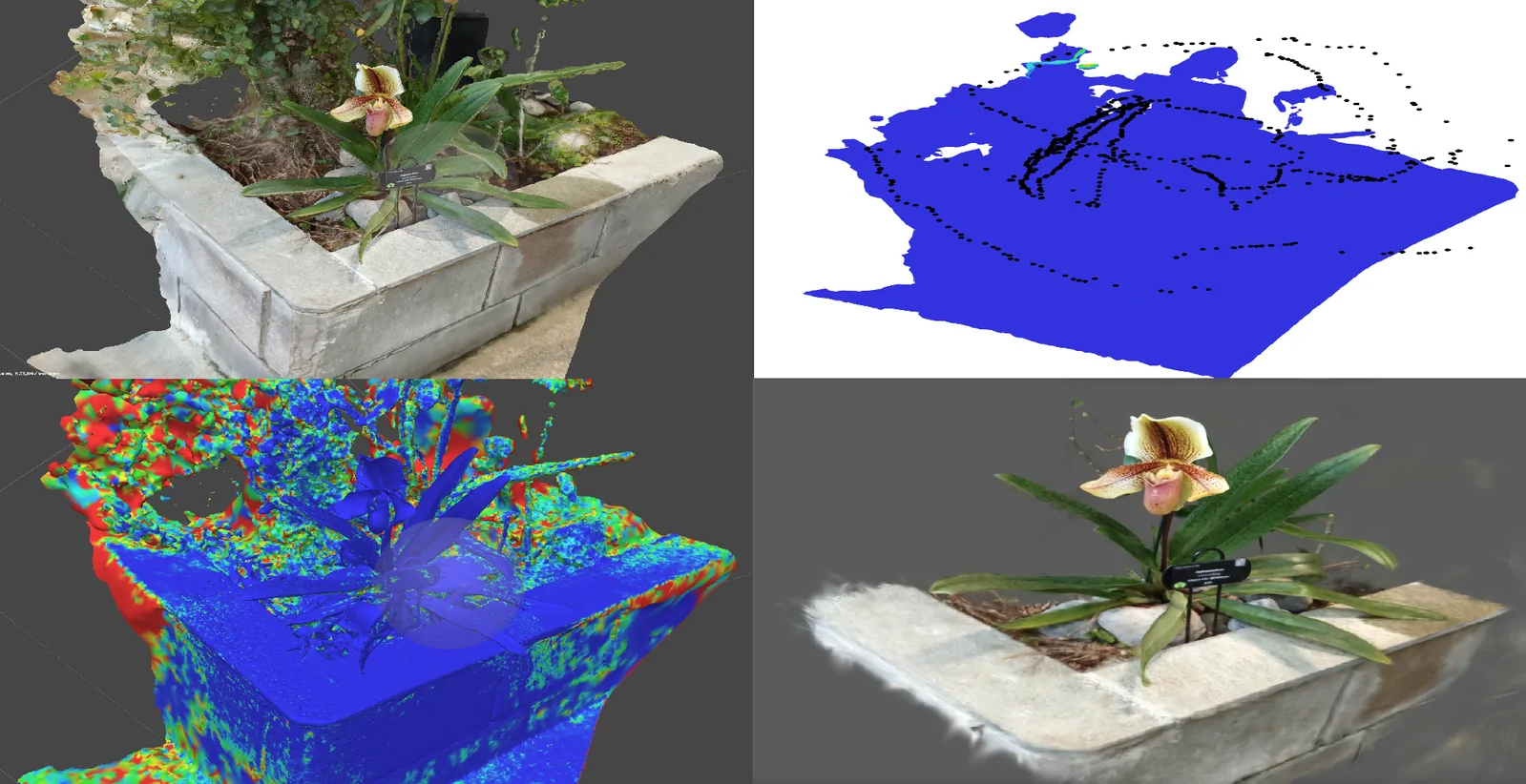
An Orchid imaged with a smartphone.

The smartphone flower demonstrated that useful reconstructions can be obtained using an entry-level smartphone despite mixed greenhouse illumination (Fig. 20).

**Figure 21.**
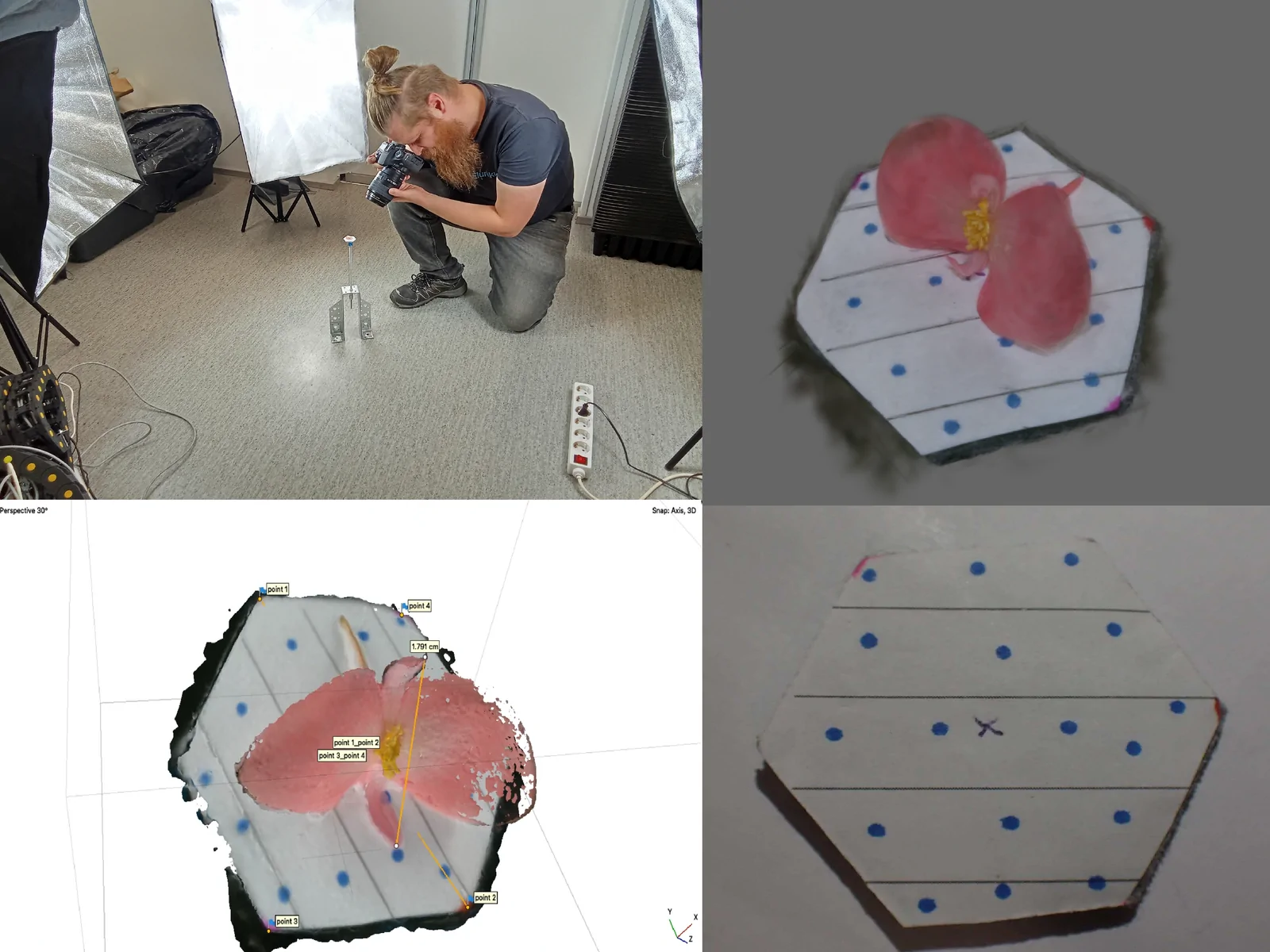
A small flower imaged using a macro objective and a marker hexagon.

**Figure 22.**
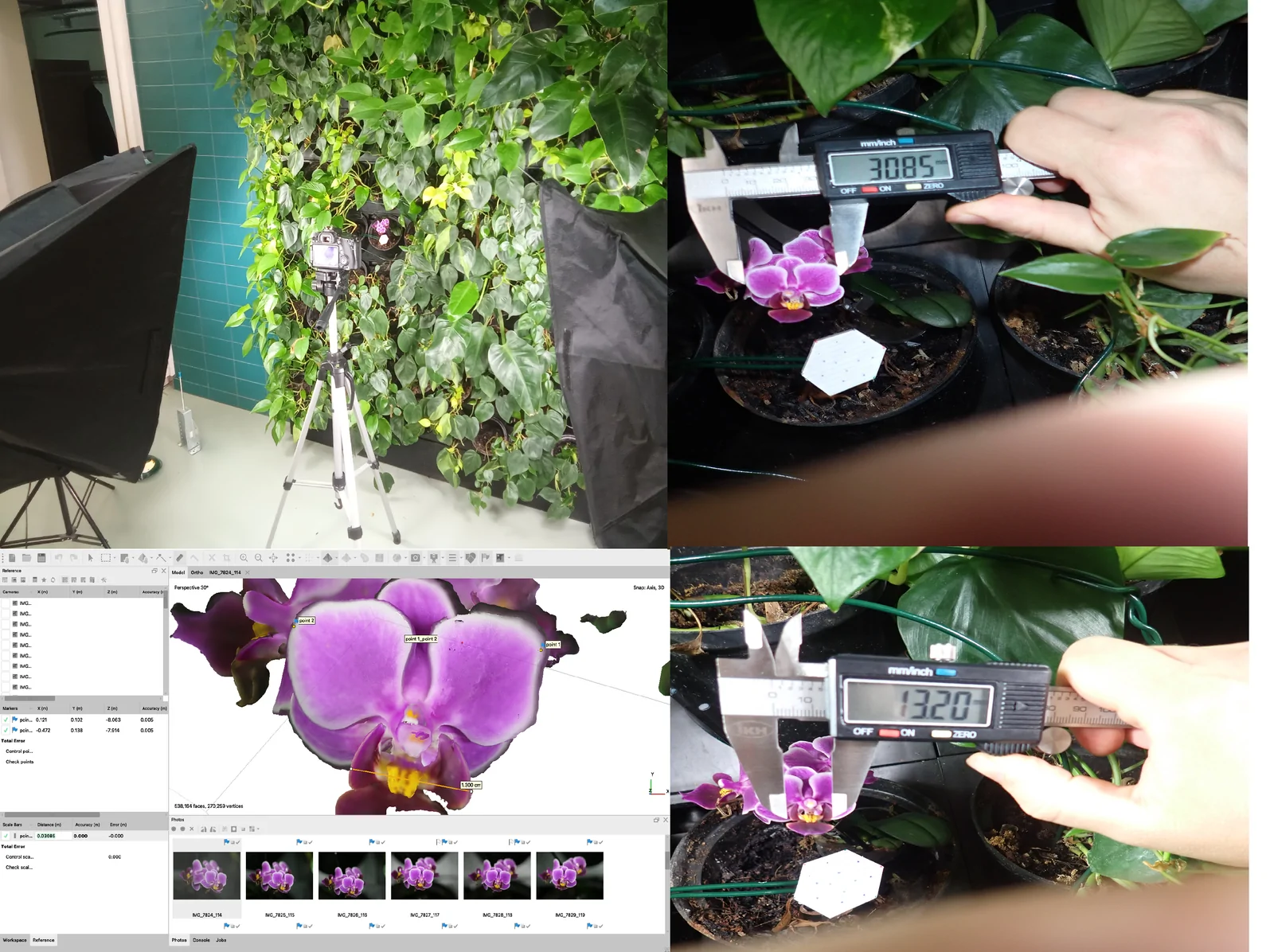
Macro measurement. Top-left: The overall imaging setting. Using a tripod and softbox lights. Top-right: Scaling measurement is taken with digital caliper. Bottom-left: Scaling measurement is added into Metashape, but other programmes can also be used. After scaling, a 3D measurement was taken (13 mm). Bottom-right: The 3D measurement was compared against digital caliper measurement (13.2 mm).

**Figure 23.**
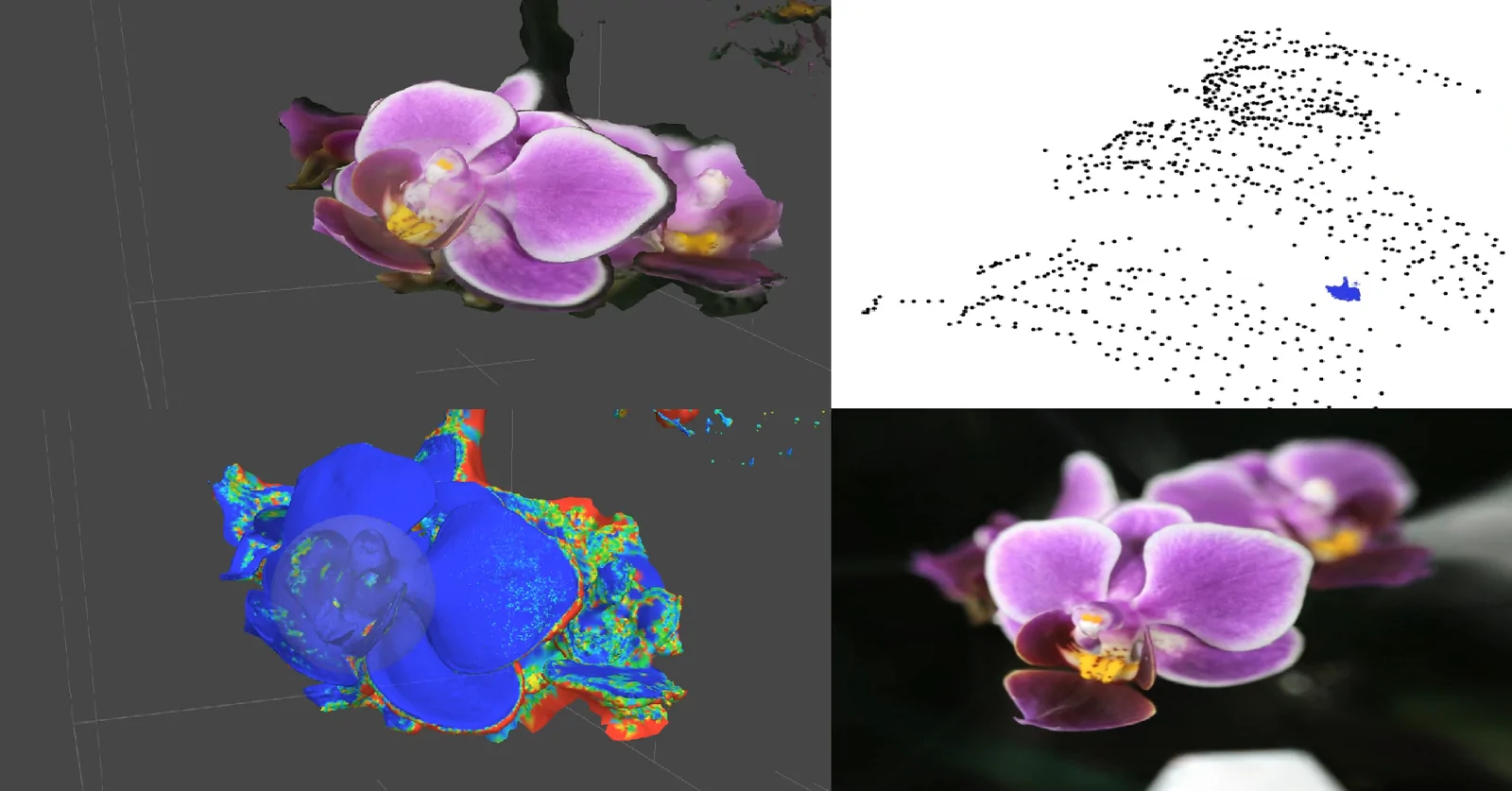
A small flower imaged from a wall, using tripod camera with a macro-lens and studio lights.

Two macro-datasets demonstrated complementary scaling approaches. MacroHexagon employed a calibrated hexagonal marker to establish scale, whereas MacroWall used a direct caliper measurement on the specimen, enabling accurate metric measurements from the reconstructed model (Figs 21, 22, 23).

**Figure 24.**
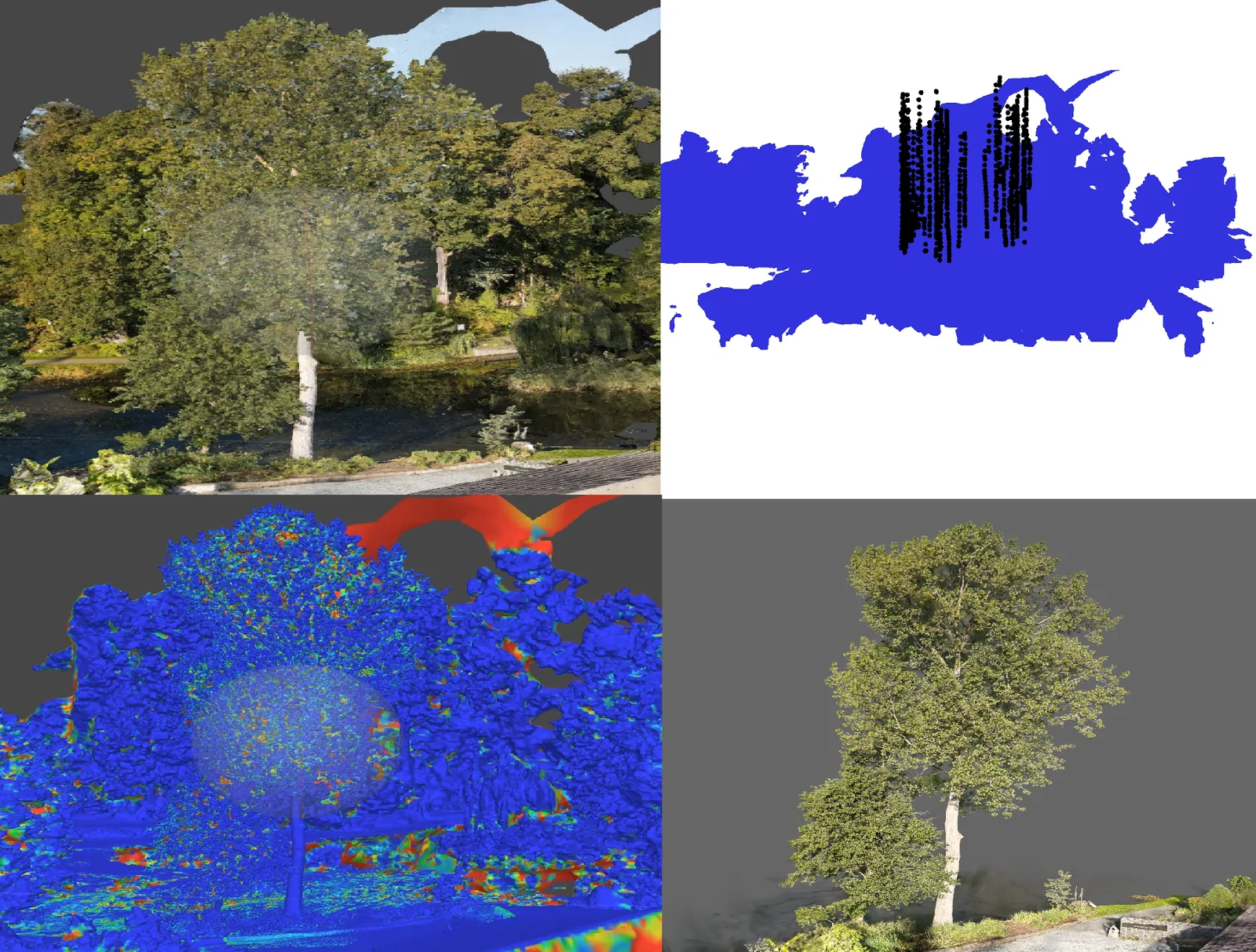
A tree imaged with a drone.

The drone-based tree was reconstructed from 1,044 aerial images acquired during a manual orbit flight. Although this dataset produced the lowest PSNR and SSIM values, it nevertheless generated a visually interpretable large-scale reconstruction suitable for interactive visualisation (Fig. 24).

## Discussion

**Practical implications for botanical digitisation**

Our results demonstrate that combining photogrammetry with 3D Gaussian Splatting provides a practical workflow for botanical digitisation across scales, from centimetre-sized flowers to greenhouse environments and trees. As the workflow relies on consumer-grade cameras and widely available software, it can be adopted by botanical gardens, museums and research groups without specialised systems.

The fern experiment demonstrates a practical approach to low-cost 4D digitisation. By aligning all image sets simultaneously before reconstructing individual time points, each model inherited a common coordinate system, enabling direct temporal visualisation and, after scaling, quantitative growth measurements. Unlike fixed-camera phenotyping systems, this approach uses stable background features rather than repeated camera positions, allowing flexible outdoor imaging.

The greenhouse reconstruction highlights the strengths of 3DGS for dense vegetation. Although the fourteen sections were assembled manually and are not intended for metric analysis, they demonstrate that large botanical environments can be reconstructed incrementally and explored interactively.

**Complementary roles of photogrammetry and 3DGS**

The two reconstruction methods serve different purposes. Photogrammetry produces explicit textured meshes suitable for morphometric measurements after scaling, whereas 3DGS provides highly realistic real-time visualisation that better preserves thin foliage and complex plant structures.

This difference was evident in the potted plant dataset, where long hanging flowers were only partially reconstructed as a mesh, but were reproduced almost completely by 3DGS. A combined workflow, therefore, exploits the strengths of both methods: photogrammetry for quantitative geometry and Gaussian splatting for visualisation, communication and digital twins.

**Reconstruction quality and limitations**

Reconstruction quality depended primarily on acquisition conditions. Controlled macro datasets produced the highest quantitative scores, whereas the wind experiment confirmed that even small leaf movements substantially reduce reconstruction quality. Capturing during calm weather is therefore recommended, while synchronised multi-camera systems could largely eliminate motion artefacts, albeit at considerably higher cost.

The drone-based tree produced the lowest reconstruction quality metrics, most likely because large outdoor vegetation presents greater challenges through self-occlusion, variable illumination and more complex imaging geometry. Larger objects are, therefore, not inherently reconstructed less accurately; rather, they are more difficult to image consistently.

Computational resources also remain a practical limitation. The global fern alignment required approximately seven hours and 48 GB RAM, indicating that large datasets may require subdivision or reduced processing settings.

**Relation to previous work and future perspectives**

Our findings are consistent with previous studies demonstrating the value of SfM photogrammetry for plant reconstruction and recent work showing the potential of 3D Gaussian Splatting for plant visualisation and digital twins. Although this study focuses on plants, similar workflows have been applied to other biological specimens, including focus-stacked insect photogrammetry, suggesting broad applicability across natural history digitisation.

Future work should investigate automated registration of greenhouse sections, quantitative validation against independent measurements and increasingly integrated photogrammetry–3DGS workflows. As imaging software and hardware improve, these approaches are likely to become routine tools for documenting living collections, supporting research and expanding public access to plant biodiversity.

## Additional 3D models and Unity visualisation Mac programme

Here are nine individual 3D Gaussian Splats, hosted in SuperSplat website.

https://superspl.at/view?id=48e6ea2a

https://superspl.at/view?id=5989e5fd

https://superspl.at/view?id=fcf9f631

https://superspl.at/view?id=3e751d3f

https://superspl.at/view?id=082ca45e

https://superspl.at/view?id=ede74a8d

https://superspl.at/view?id=f7067e39

https://superspl.at/view?id=869ead7d

https://superspl.at/view?id=70a1a6ba

Unity game engine visualisation. Apple Silicon version.

GreenhouseMac

https://youtu.be/e-gOYYoLn0E?si=MJJISgdUxDdOE6vE

Tree model 3DGS:

https://superspl.at/view?id=b9710f84

Tree before trimming 3DGS:

https://superspl.at/view?id=af75a380

Macro flower 3DGS:

https://superspl.at/view?id=4cfbc0ff

Orchid 3DGS scanned with a smartphone:

https://superspl.at/view?id=3badb8f4

Potted plant: https://youtu.be/ha3fxTKUC8I?si=LTIYG4hEOJi7Qcrr

## Conclusions

Photogrammetry and 3D Gaussian Splatting enable affordable, visually rich 3D digitisation of plants across scales, supporting both quantitative morphology workflows and interactive visualisation. We demonstrate a 12-timepoint fern sequence aligned in a shared coordinate system and a multi-part 3DGS digital twin of a tropical greenhouse assembled in Unity, along with additional macro, smartphone and drone-based examples. While photogrammetry remains preferable for metric geometry and trait extraction, 3DGS offers compelling real-time visualisation for occluded, thin-structure vegetation scenes. We encourage botanical institutions and researchers to adopt and share similar 3D digitisation workflows to improve the reachability, preservation and communication of living vegetation.

## Funding

This study has been funded by The University of Tartu / The University of Tartu Natural History Museum. This research has been financed by the Estonian Research Council [PRG1170], the Estonian research infrastructure roadmap project NATARC and by the Ministry of Education and Research through the Estonian Research Council (Center of Excellence AgroCropFuture “Agroecology and new crops in future climates”, project TK200). The funders had no role in study design, data collection and analysis, decision to publish, or preparation of the manuscript.

## Figures and Tables

**Figure 2.** Video demonstrating the photogrammetric workflow used to generate the 12-timepoint ("4D") fern reconstruction. **00:04**: All images from the twelve acquisition sessions are first aligned in a single Agisoft Metashape project to establish a shared camera network and coordinate system. The project is then duplicated and each copy reduced to a single imaging day by removing photographs from the remaining timepoints, while preserving the camera alignment. Consequently, all daily reconstructions inherit the same orientation and, after scaling the original project, the same metric coordinate system. Stable background features surrounding the fern provide the common reference for alignment; **02:16**: Reconstructed camera positions illustrating the hand-held image acquisition using an Olympus TG-6 (12 MP); **06:33**: Global multi-day alignment in Metashape. The alignment of approximately 8,500 images required 48 GB RAM using Low alignment accuracy. For larger datasets or systems with limited memory, the initial alignment could potentially be divided into overlapping subsets that share one common timepoint, followed by chunk alignment to establish a common coordinate system; **08:10**: VisualSFM visualisation of incremental sparse point cloud generation. Although VisualSFM is no longer used for the primary processing, it provides an intuitive illustration of Structure-from-Motion reconstruction; **09:28**: Individual daily camera alignments exported from Metashape in COLMAP format and reconstructed as 3D Gaussian Splatting models in Brush v.0.3. The resulting Gaussian splats are visualised in SuperSplat.

**Figure 5.**
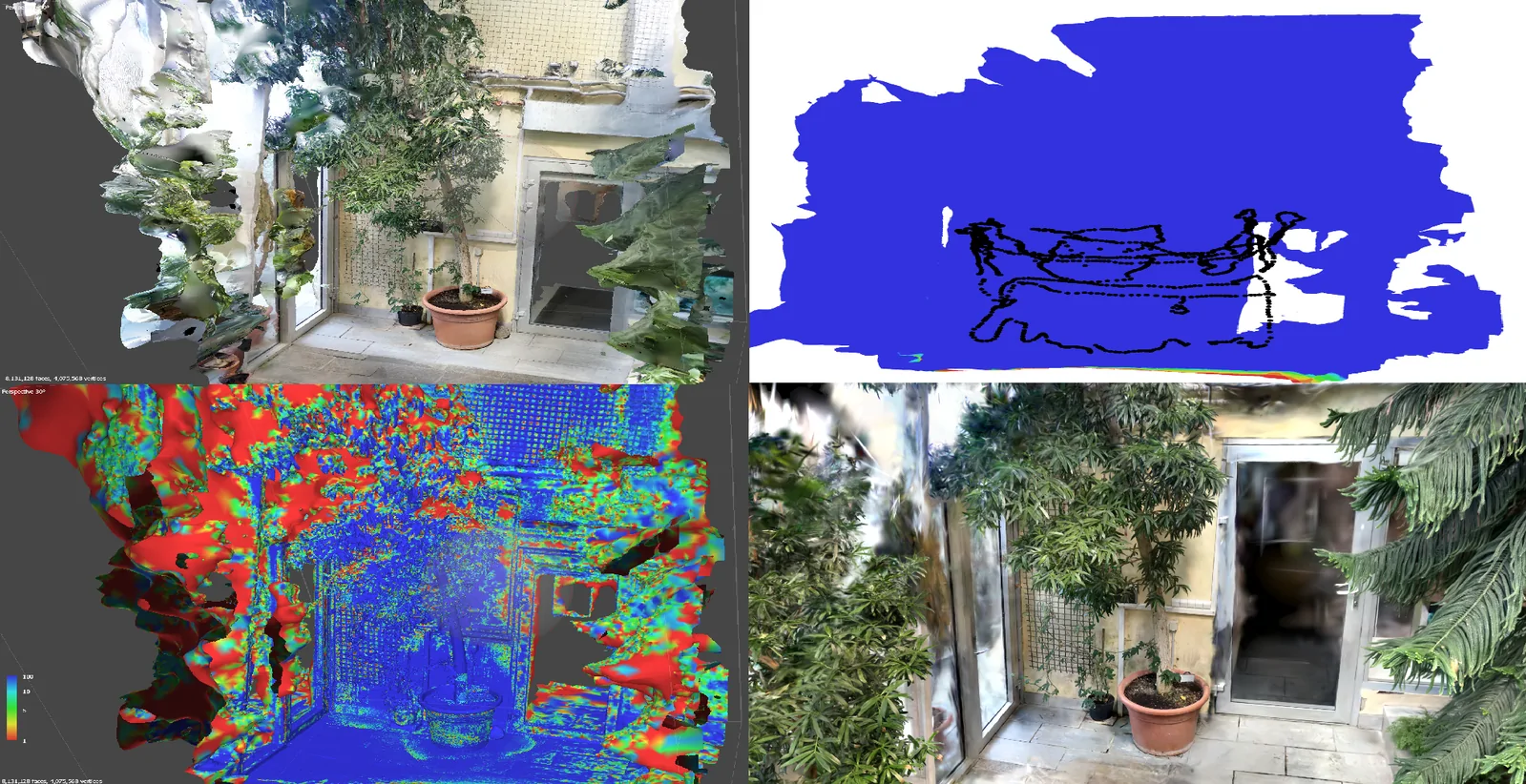
Position 1. Top-left: Textured 3D model in Metashape. Top-right: camera positions. Bottom-left: Meshconfidence level in Metashape. Bottom-right: 3D Gaussian Splat.

**Figure 6.**
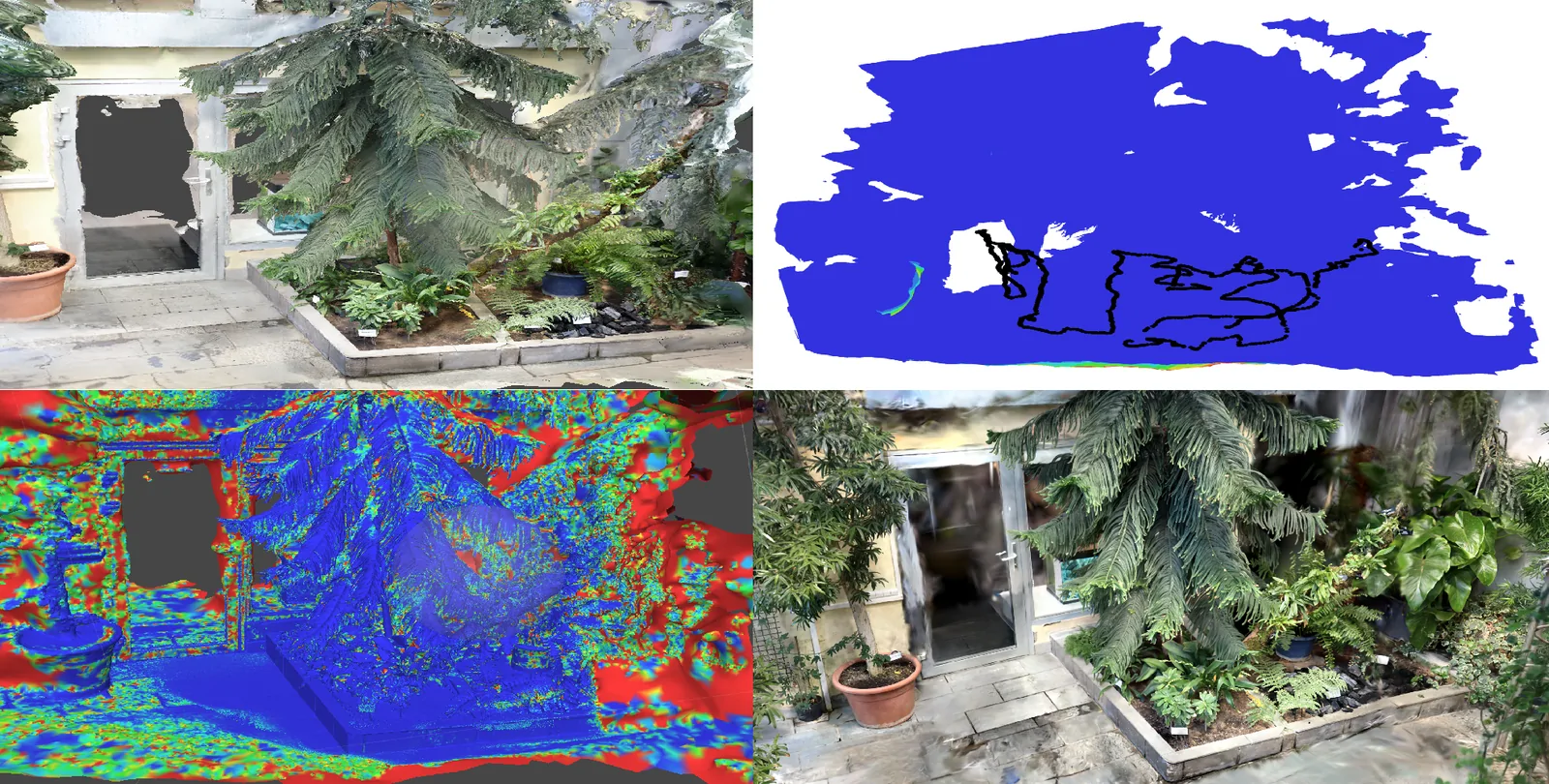
Position 2.

**Figure 7.**
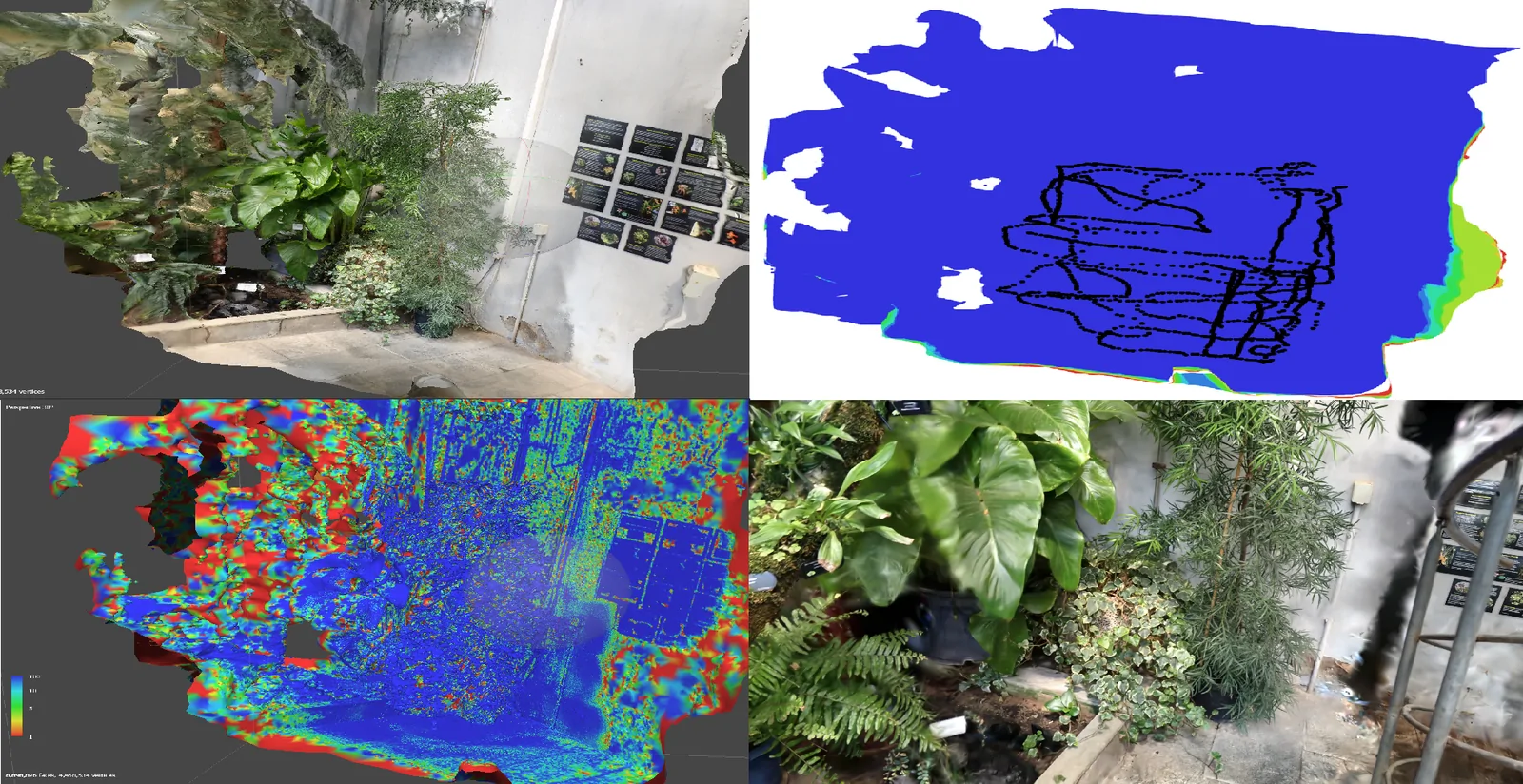
Position 3.

**Figure 8.**
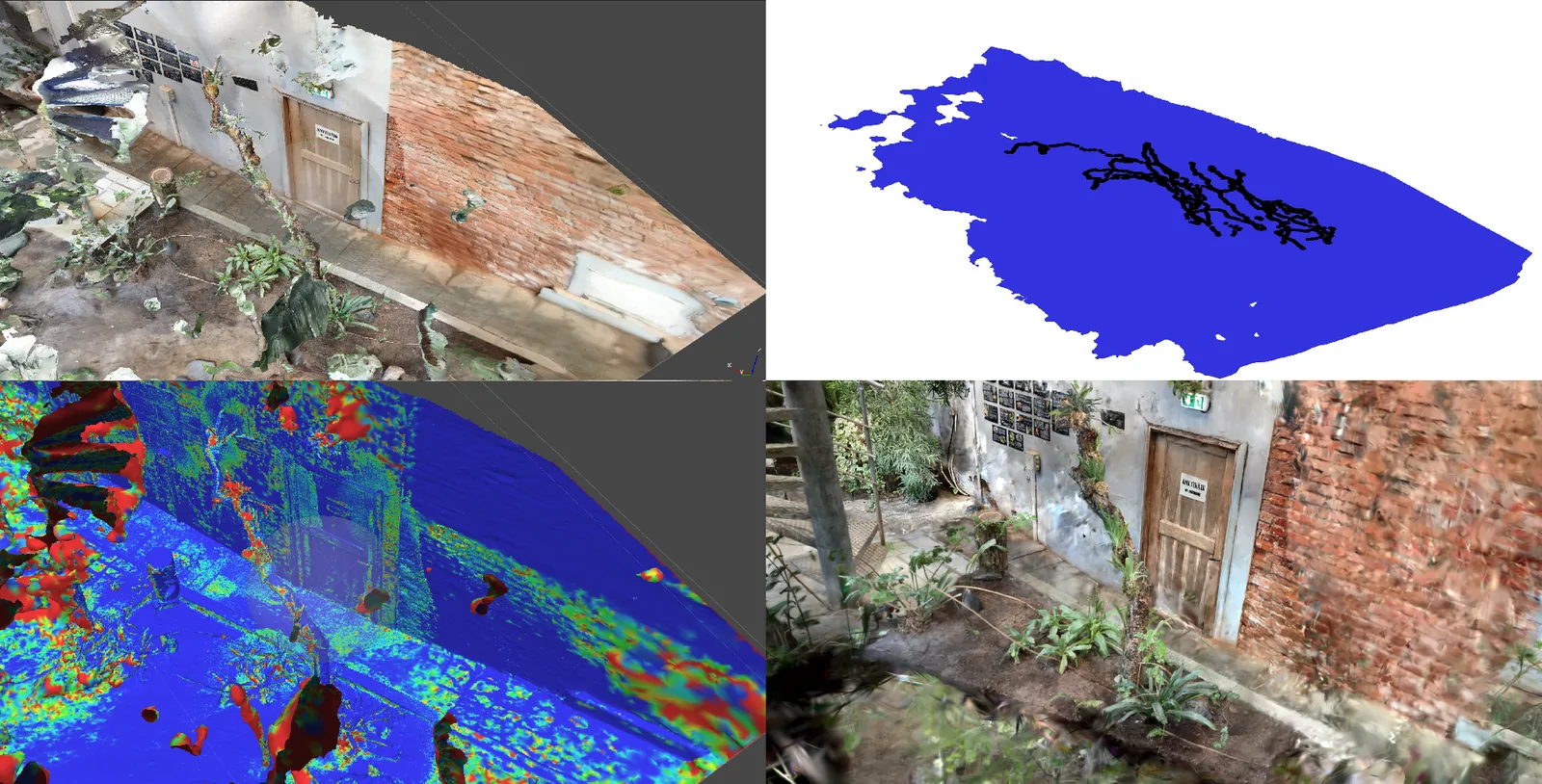
Position 4.

**Figure 9.**
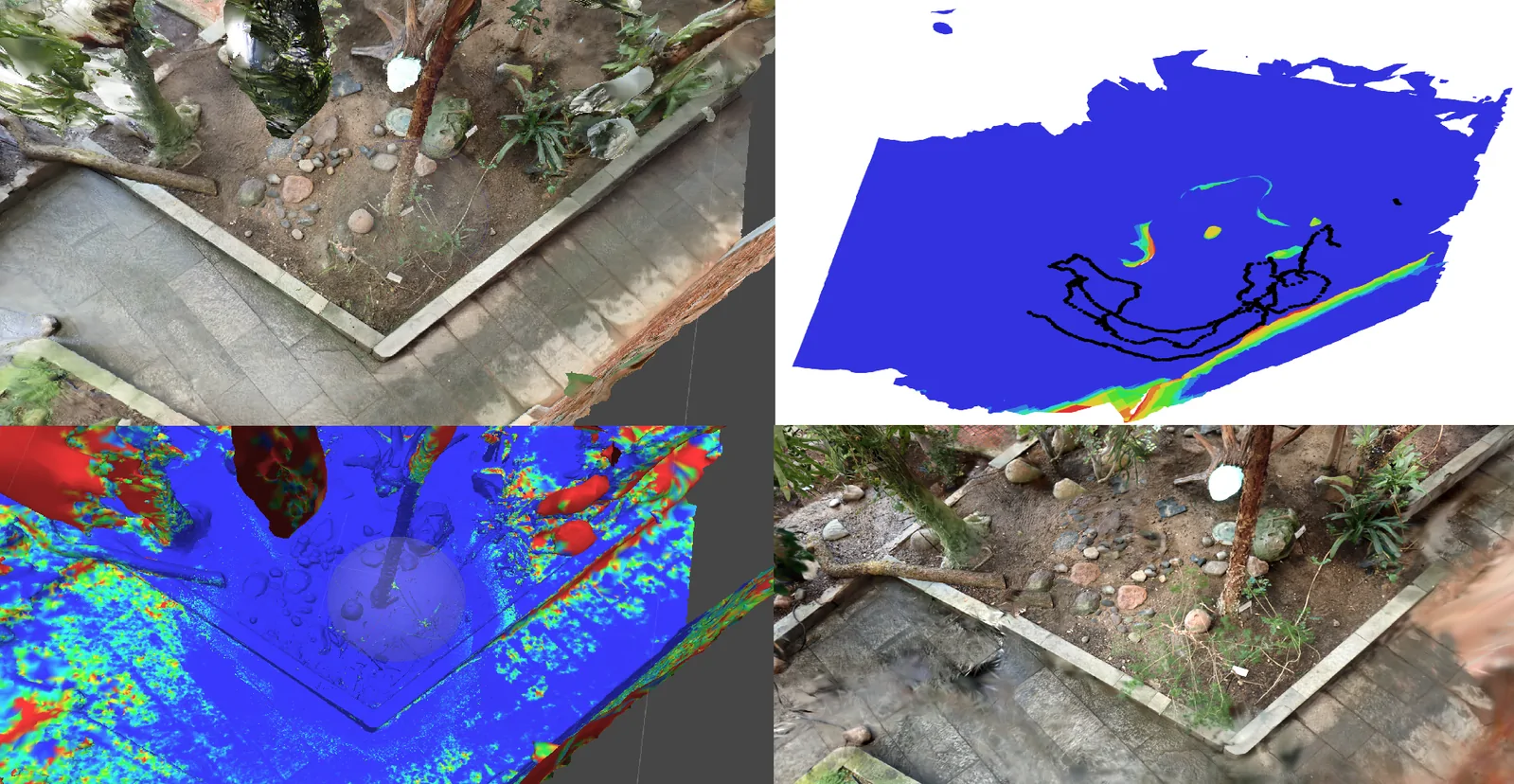
Position 5.

**Figure 10.**
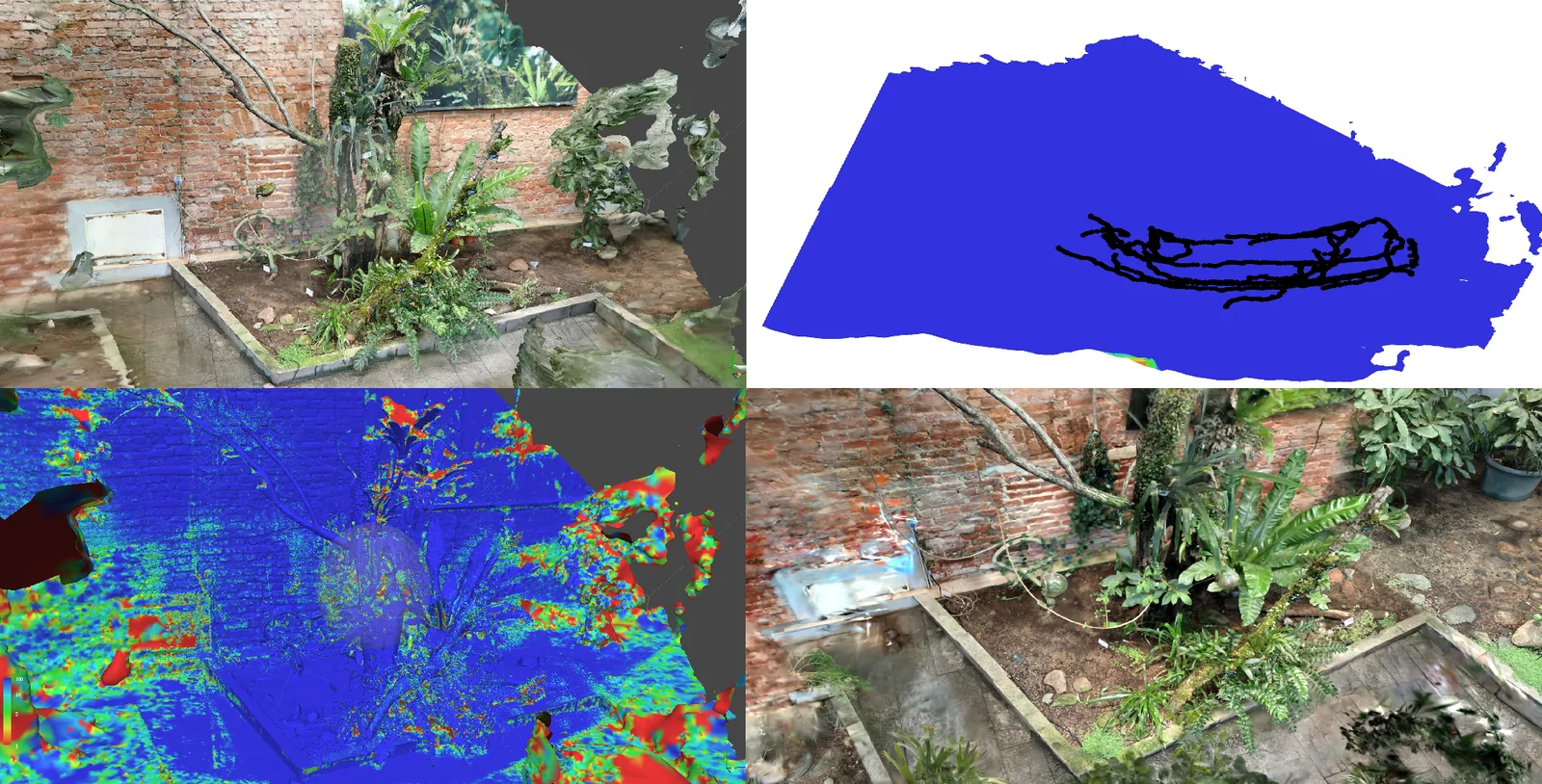
Position 6.

**Figure 11.**
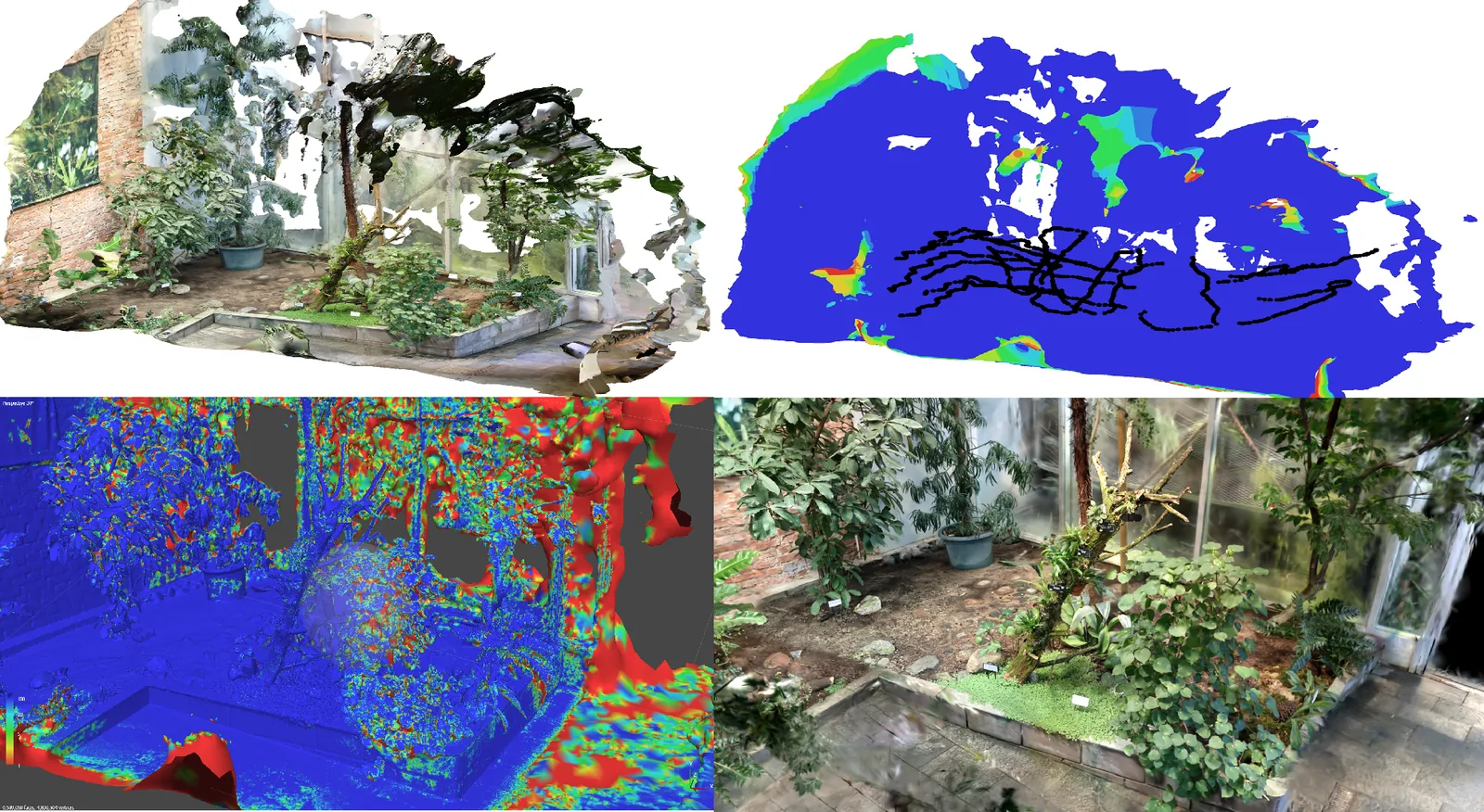
Position 7.

**Figure 12.**
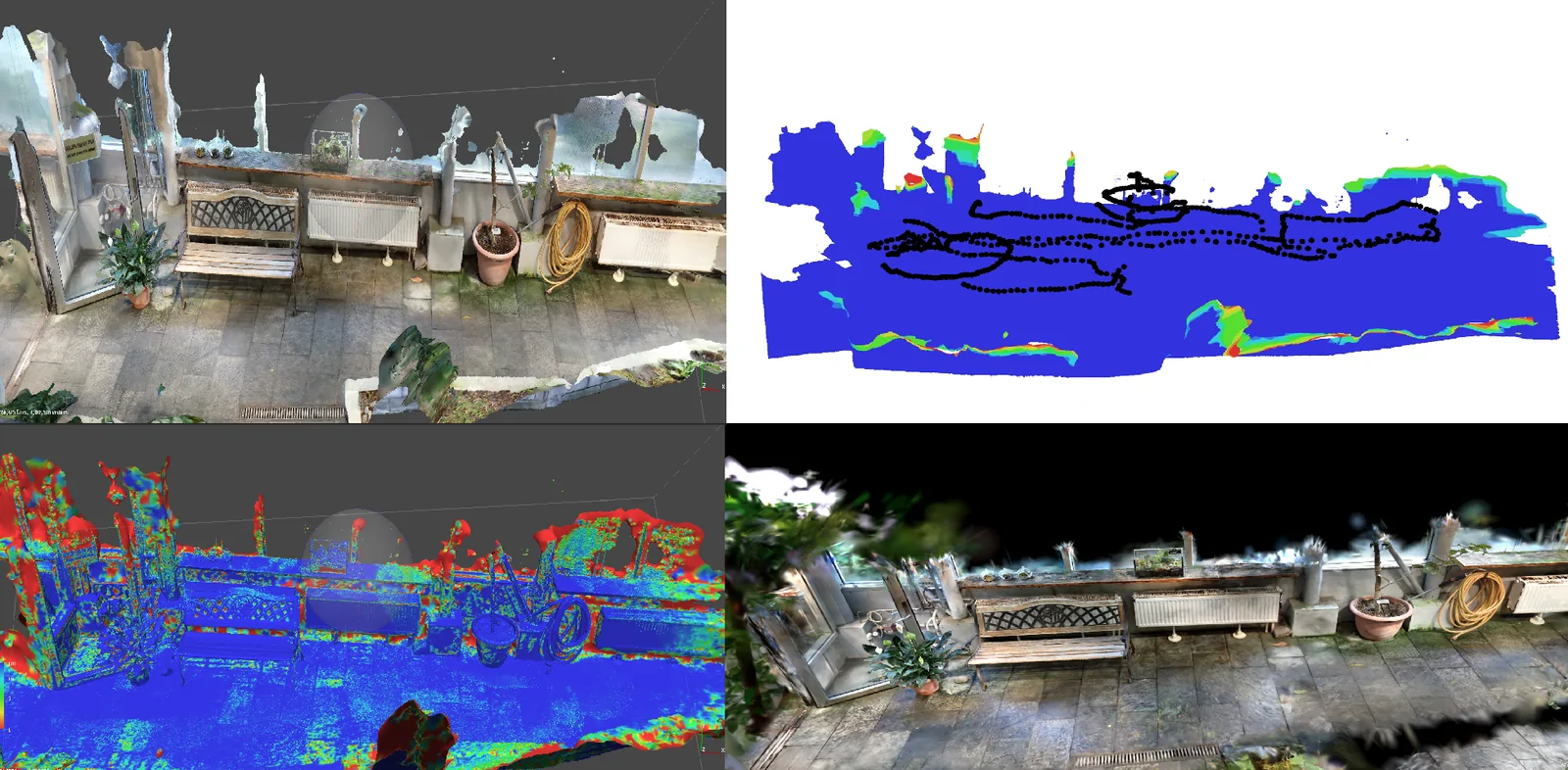
Position 8.

**Figure 13.**
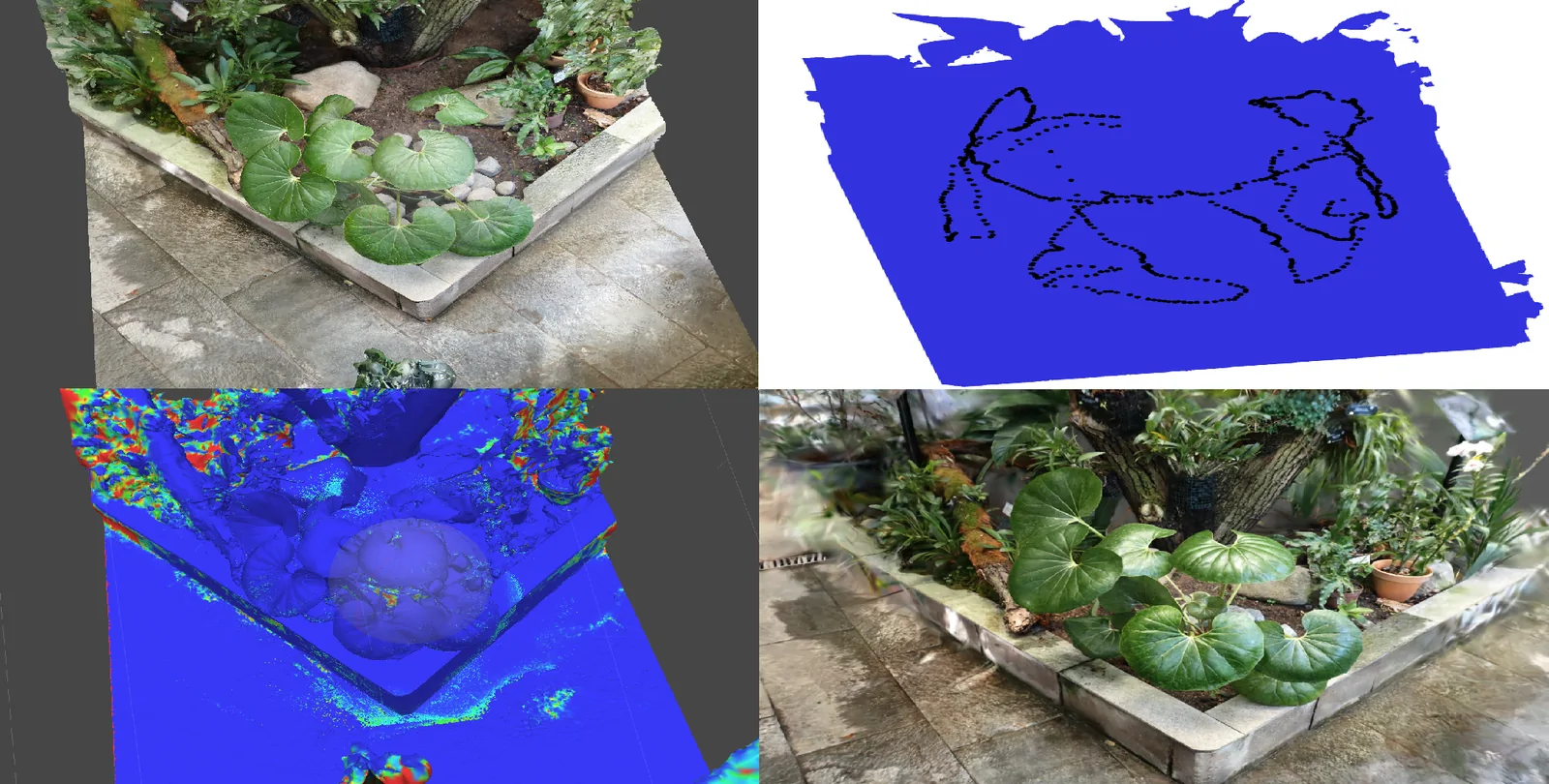
Position 9.

**Figure 14.**
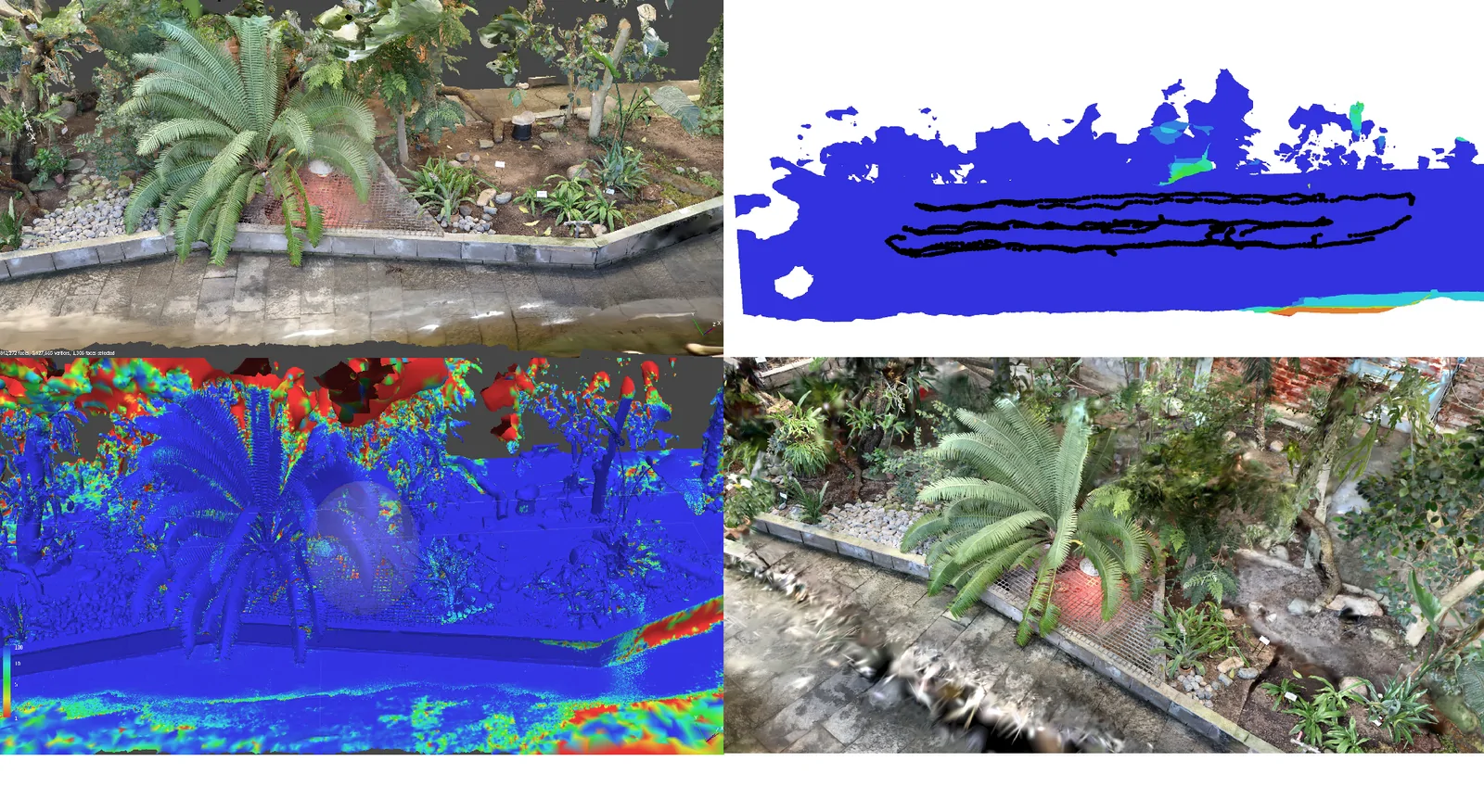
Position 10.

**Figure 15.**
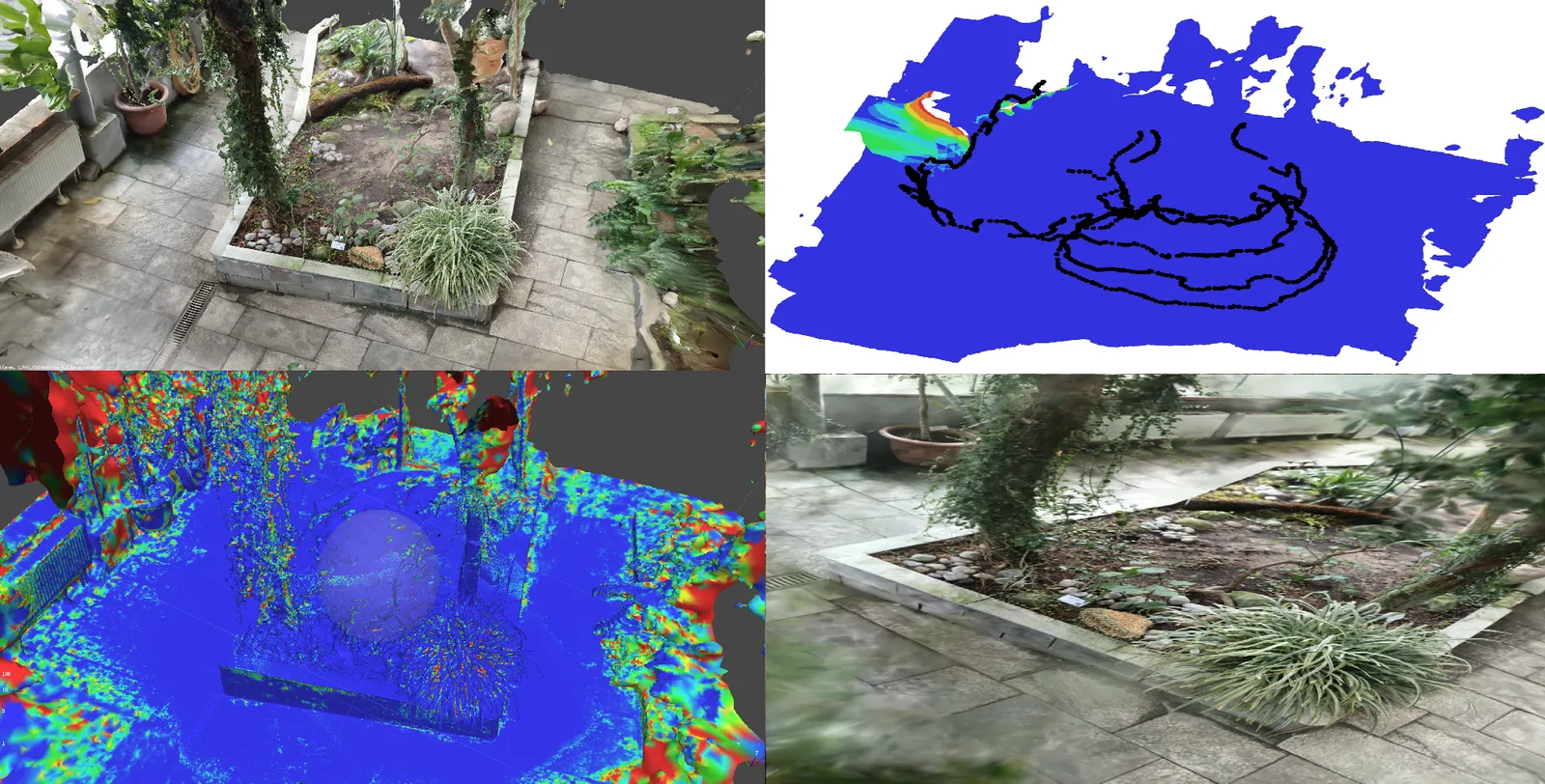
Position 12.

**Figure 16.**
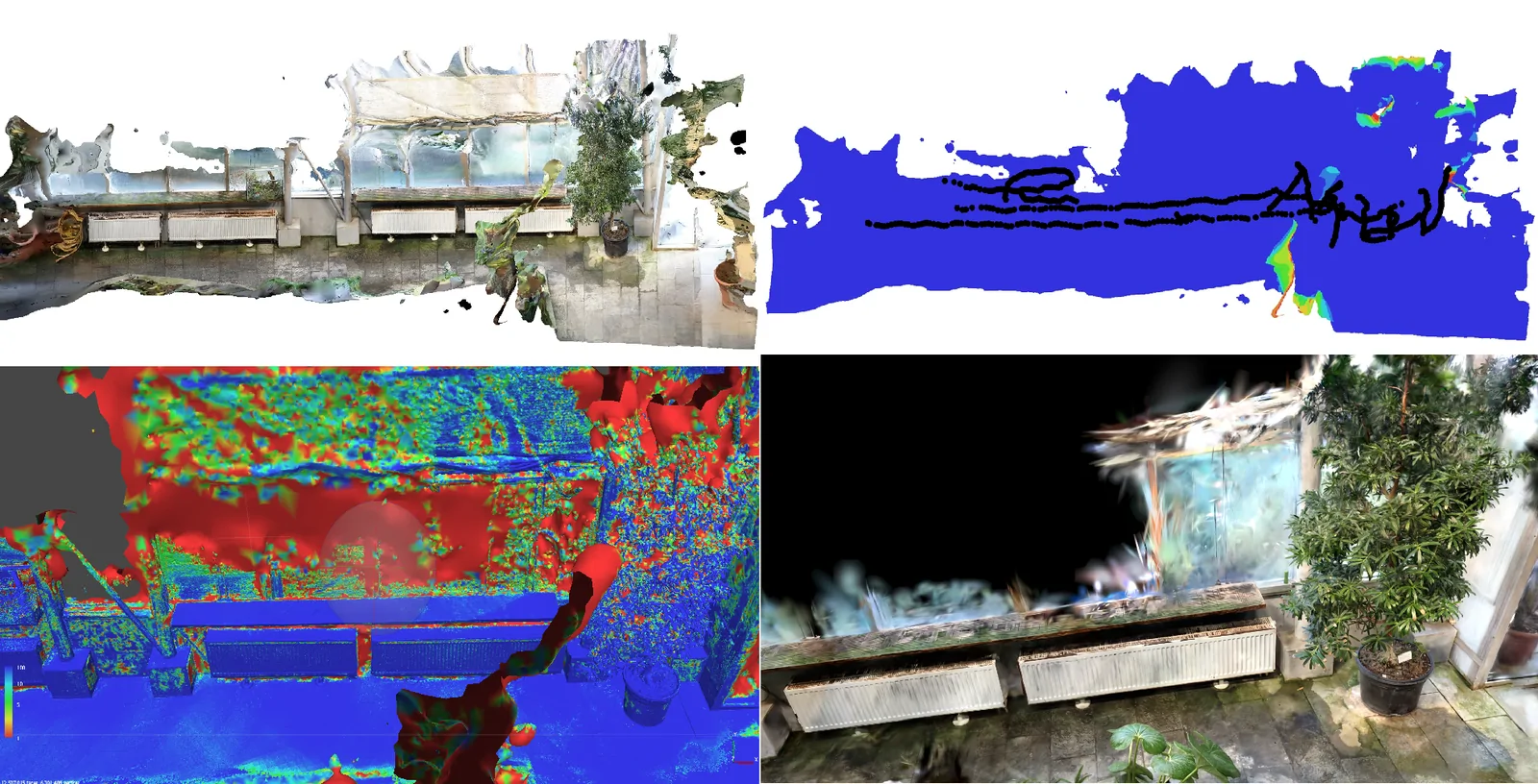
Position 13.

**Table 1.** Dataset summary and intended use.

Dataset / case	Site / context	Sensor	Images (n)	Notes on scale / intended use
Fern time series (12 timepoints)	Outdoor botanical garden	Olympus TG-6 (12 MP)	~8,500 total; 300–1,200 per day	Scaled using measured site distances; metric time-series possible
Greenhouse digital twin (14 sections)	Tropical greenhouse	Olympus TG-6 (12 MP)	~20,000 total; ~500–1,500 per section	Sections assembled manually in SuperSplat/Unity; visualization-first (not metrically registered)
Potted plant	Indoor	Olympus TG-6 (12 MP)	1,575	Raised stand improved low-angle coverage
Smartphone flower	Greenhouse	Xiaomi Redmi A3 (8 MP)	700	Low-cost capture; mixed lighting
MacroWall	Indoor plant wall	Canon 70D + Canon 100 mm macro (1:1)	682	Portable studio lighting; in-situ macro capture
MacroHexagon	Controlled macro setup	Canon 70D + Canon 100 mm macro (1:1)	360	Portable studio lighting; direct caliper-based scaling
Drone tree	Outdoor	DJI Mavic Air 2	1,044	Manual orbit flight; landscape-scale demonstration
Wind test (no wind / wind)	Indoor	Olympus TG-6 (12 MP)	667 / 670	Controlled comparison of motion effects on reconstruction quality

**Table 2.** Reconstruction quality metrics for the twelve fern imaging sessions. Day numbers correspond to acquisition days within the fifteen-day observation period; not every calendar day was imaged.

Time point (day)	Images	Tie Points	RMS reprojection error (px)	PSNR (dB)	SSIM	LPIPS
1	576	178,52	1.56	25.1126	0.8215	0.2824
2	774	207,611	1.53	25.6842	0.8386	0.2599
3	499	157,144	1.49	26.036	0.8296	0.2933
4	544	156,797	1.51	26.0997	0.818	0.298
5	834	236,22	1.47	25.8312	0.8024	0.3239
6	712	197,342	1.51	25.5046	0.8424	0.2231
8	501	165,415	1.50	25.5205	0.7875	0.3388
9	341	95,698	1.36	26.3541	0.8293	0.2687
10	710	189,761	1.47	21.9987	0.726	0.3248
12	875	244,542	1.59	25.3838	0.7938	0.3187
13	1,204	325,415	1.63	24,06	0.781	0.3288
15	969	254,017	1.72	24.211	0.7745	0.3106

**Table 3.** Fourteen independently reconstructed greenhouse sections.

Greenhouse position	Images	Tie Points	RMS reprojection error (px)	PSNR (dB)	SSIM	LPIPS
1	1350	795,675	2.35	23.4997	0.8239	0.3898
2	1143	865,186	2.08	23.1173	0.7173	0.4666
3	1643	1,115,847	2.10	23.0893	0.7757	0.4004
4	978	668,859	2.23	24.2336	0.7902	0.4918
5	768	518,496	2.19	23.7509	0.719	0.5199
6	1320	854,012	2.32	22.8967	0.7453	0.5202
7	1187	745,483	2.02	24.8066	0.7926	0.424
8	708	509,839	2.30	25.6637	0.8458	0.4384
9	710	329,238	1.64	26.2591	0.8343	0.3822
10	1039	520,519	1.49	26.0945	0.8362	0.3102
11	940	594,01	2.15	24.4411	0.7517	0.4715
12	1194	811,243	2.20	23.331	0.737	0.4892
13	782	442,796	1.53	25.4936	0.8459	0.3356
14	546	354,318	2.16	21.4913	0.695	0.5017

**Table 4.** Individual datasets.

phone flower	669	438,176	2.76	23.9856	0.7741	0.4634
potted plant	1,568	680,635	3.42	25.9269	0.8939	0.3416
drone tree	1,044	653,742	0.713	17.3731	0.5328	0.4191
fernNoWind	667	363,512	2.17	29.7445	0.9154	0.2801
fernWithWind	670	346,927	2.48	25.4135	0.8627	0.3486
macroWall	682	179,165	3.27	23.0407	0.8527	0.2734
macroHexagon	360	76,236	4.09	24.9369	0.9032	0.1346
